# Resolving Photoinduced Femtosecond Three-Dimensional Solute–Solvent Dynamics through Surface Hopping Simulations

**DOI:** 10.1021/acs.jctc.4c00169

**Published:** 2024-05-20

**Authors:** Severin Polonius, David Lehrner, Leticia González, Sebastian Mai

**Affiliations:** † Institute of Theoretical Chemistry, Faculty of Chemistry, University of Vienna, Währinger Str. 17, 1090 Vienna, Austria; ‡ Vienna Doctoral School in Chemistry (DoSChem), University of Vienna, Währinger Str. 42, 1090 Vienna, Austria; § Vienna Research Platform on Accelerating Photoreaction Discovery, University of Vienna, Währinger Straße 17, 1090 Vienna, Austria

## Abstract

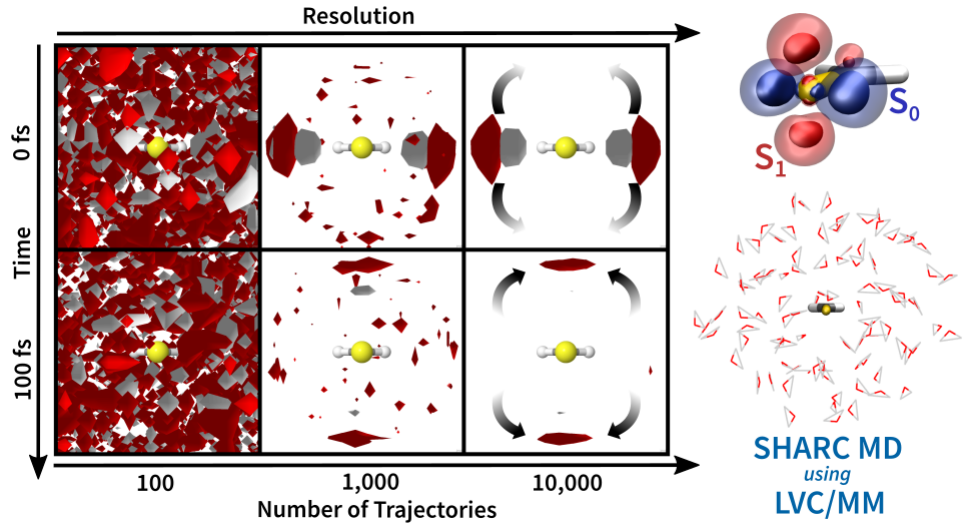

Photoinduced dynamics in solution is governed by mutual solute–solvent interactions, which give rise to phenomena like solvatochromism, the Stokes shift, dual fluorescence, or charge transfer. Understanding these phenomena requires simulating the solute’s photoinduced dynamics and simultaneously resolving the three-dimensional solvent distribution dynamics. If using trajectory surface hopping (TSH) to this aim, thousands of trajectories are required to adequately sample the time-dependent three-dimensional solvent distribution functions, and thus resolve the solvent dynamics with sub-Ångstrom and femtosecond accuracy and sufficiently low noise levels. Unfortunately, simulating thousands of trajectories with TSH in the framework of hybrid quantum mechanical/molecular mechanical (QM/MM) can be prohibitively expensive when employing ab initio electronic structure methods. To tackle this challenge, we recently introduced a computationally efficient approach that combines efficient linear vibronic coupling models with molecular mechanics (LVC/MM) via electrostatic embedding [Polonius et al., JCTC **2023,***19,* 7171–7186]. This method provides solvent-embedded, nonadiabatically coupled potential energy surfaces while scaling similarly to MM force fields. Here, we employ TSH with LVC/MM to unravel the photoinduced dynamics of two small thiocarbonyl compounds solvated in water. We describe how to estimate the number of trajectories required to produce nearly noise-free three-dimensional solvent distribution functions and present an analysis based on approximately 10,000 trajectories propagated for 3 ps. In the electronic ground state, both molecules exhibit in-plane hydrogen bonds to the sulfur atom. Shortly after excitation, these bonds are broken and reform perpendicular to the molecular plane on timescales that differ by an order of magnitude due to steric effects. We also show that the solvent relaxation dynamics is coupled to the electronic dynamics, including intersystem crossing. These findings are relevant to advance the understanding of the coupled solute–solvent dynamics of solvated photoexcited molecules, e.g., biologically relevant thio-nucleobases.

## 1 Introduction

The intricate interplay between solute and solvent molecules is key to understand solubility, structure, (catalytic) reactivity, as well as molecular dynamics and the nature of electronic states.^1−5^ Importantly, solvent molecules do not only exert electrostatic influence on a solute molecule and its electronic states but are also reciprocally influenced by the electronic structure of the solute. This is particularly true for photoinduced processes where excited electronic states can exhibit drastically different electrostatics compared to the ground state and to each other.^6^ These differences in electrostatics are at the origin of phenomena like solvatochromism, the Stokes shift,^7,8^ solvent-dependent dual fluorescence,^9,10^ or solvent-dependent charge transfer dynamics.^11,12^ All of these phenomena involve complex entangled dynamics of solute and solvent involving all of their nuclear and electronic degrees of freedom.

The experimental observation of ultrafast coupled solute–solvent dynamics is very challenging and often requires the combination of multiple techniques such as time-resolved X-ray solution scattering,^12−15^ transient absorption spectroscopy,^16,17^ or X-ray fluorescence spectroscopy.^18−20^ These challenges make computational simulations essential in the analysis and interpretation of such experiments. These simulations should ideally be able to describe (i) the nonadiabatic dynamics of a solute molecule, (ii) the mutual interaction of solute and solvent molecules with (iii) femtosecond time resolution but simultaneously on (iv) sufficiently long timescales, and with (v) sufficiently low noise levels of the (three-dimensional) solvent distribution functions at an adequate spatial resolution. The requirements (i)–(iii) can be met by nonadiabatic molecular dynamics simulations,^21−25^ for instance with trajectory surface hopping (TSH)^26−28^ in a quantum mechanical/molecular mechanics (QM/MM) framework, i.e., employing a quantum-mechanical description of the solute coupled with a classical solvent.^29−31^ In contrast, the requirements (iv) and (v) pose a significant challenge due to the high computational cost associated with ab initio TSH simulations. While current state-of-the-art (solute-focused) TSH simulations are typically limited to a few hundred trajectories^23^ and propagation times of a few picoseconds, a detailed description of solute–solvent dynamics would require thousands of trajectories (vide infra) and propagation up to tens or even hundreds of picoseconds.^32,33^

Recently, we have presented a novel approach to perform solvation-focused TSH simulations through the combination of a linear vibronic coupling (LVC) model^34^ with a classically described solvent in a QM/MM fashion, using electrostatic embedding based on distributed multipoles for the solute.^35^ This approach, called LVC/MM, is an extension of the LVC model approach that has previously been shown to enable highly efficient TSH simulations within the surface hopping including arbitrary coupling (SHARC)^36−39^ program package.

**Figure 1:**
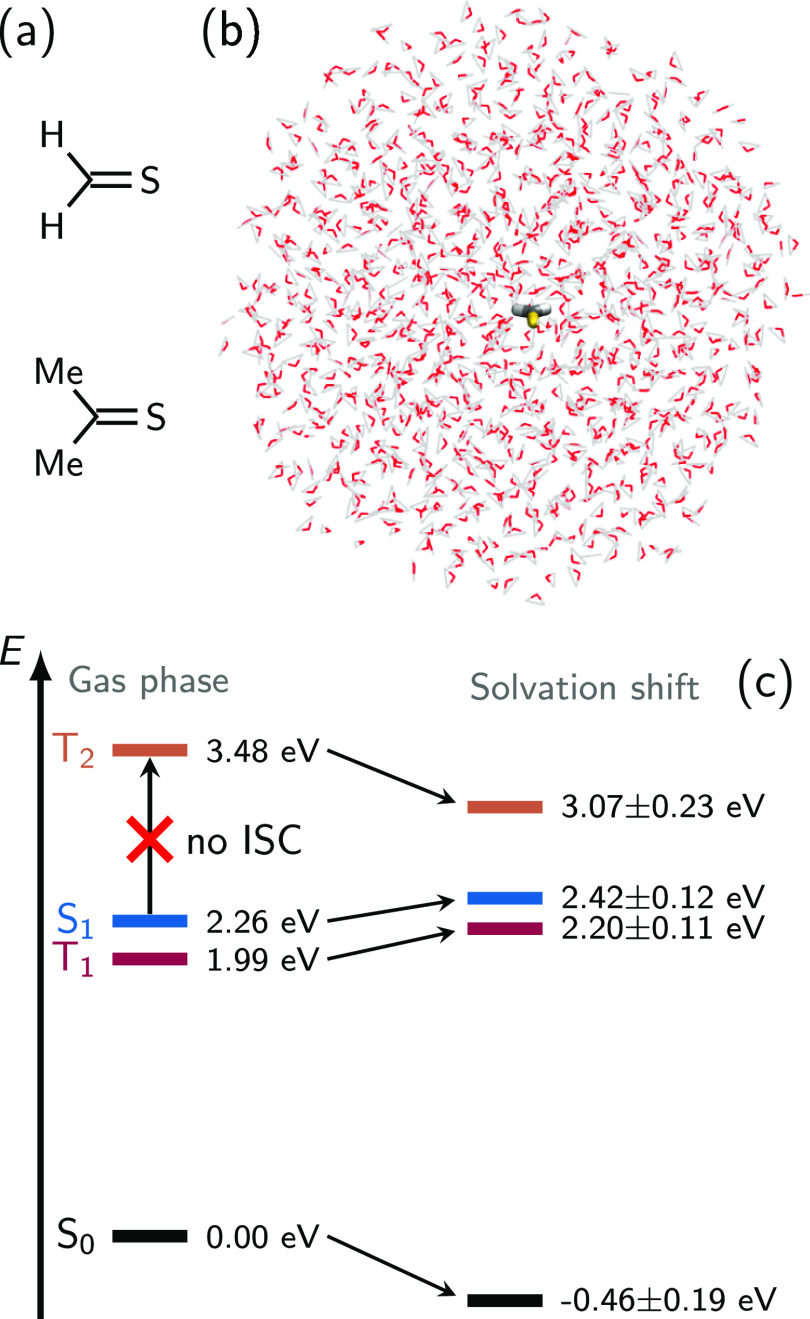
(a) Structures of CH_2_S and CMe_2_S, (b) simulated water droplet with CH_2_S, and (c) equilibrium energies for the gas phase and respective solvation shift for CH_2_S. The gas-phase energies are taken from the LVC parameters (obtained at the MS-CASPT2 level of theory). The solvation shifts are calculated from averages and standard deviations from an equilibrated LVC/MM trajectory in the S_0_ state in solution and include the electrostatic embedding term. The state characters are discussed in Section 5.1.

The goal of this work is to demonstrate how the LVC/MM method facilitates the simulation of the coupled evolution of the solute’s nuclei and electrons—including intersystem crossing (ISC)—and the three-dimensional solvent distribution, with a simultaneous sub-Å and femtosecond accuracy. The method is applied to unravel the solute–solvent relaxation dynamics of thioformaldehyde (CH_2_S) and thioacetone (CMe_2_S) in water (Figure 1a,b). Although these compounds are highly reactive and short-lived in solution and in the gas phase, where they oligomerize within seconds,^40^ they still constitute useful test systems for three reasons: (i) CH_2_S is the smallest thiocarbonyl and has been previously used to benchmark the performance of different electronic structure methods and decoherence schemes in TSH,^41,42^ as well as to demonstrate the capabilities of the LVC/MM method in the ground state.^35^ The present study adds thioacetone (CMe_2_S) as a heavier analogue to investigate the effect of steric hindrance. (ii) The nonadiabatic dynamics of both molecules is governed by the ^1^nπ*–^3^ππ* (S_1_–T_2_) energy gap,^43−45^ which is too large to allow fast ISC^41^ in the gas phase but is dynamically modulated in aqueous solution by hydrogen bonds^35^ (Figures 1c and S1). (iii) Both molecules can serve as prototypes to understand solute–solvent dynamics of larger molecules containing thiocarbonyl groups, e.g., biologically relevant thionated nucleobase analogues.^46−50^

In the following, we outline the core equations of the LVC/MM method (Section 2) and describe the computations in terms of the LVC model parametrization, system preparation with MM, and nonadiabatic dynamics using SHARC (Section 3). Importantly, in Section 4, we discuss novel aspects of the analysis of temporally and spatially resolved solvent distributions and provide guidelines for choosing the number of trajectories depending on the investigated processes. Finally, in Section 5, we analyze the results obtained for both thiocarbonyls.

## 2 Theory

Here, we only provide the essential equations of electrostatic embedding LVC/MM—the method is fully documented in ref (35). The solute is described by the LVC Hamiltonian matrix **H**_LVC_(**R**_QM_), which is constructed from coupled harmonic oscillators in normal-mode coordinates for all diabatic states. In LVC/MM, these diabatic states are electrostatically coupled to a set of point charges via the interaction matrix **X**
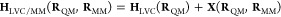
1

The interaction term *X*_*ij*_ of two states *i* and *j* is given by
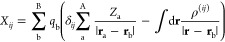
2where *q*_b_ and **r**_b_ are charges and positions of the solvent atoms, *Z*_a_ and **r**_a_ are charges and positions of the solute atoms, and ρ^(*ij*)^ is an electron density (*i* = *j*) or transition density (*i* ≠ *j*). The term *X*_*ij*_ describes solvent-induced shifts in energy (*i* = *j*) and solvent-induced state mixing (*i* ≠ *j*).

LVC models typically do not carry explicit information about the electronic wave functions (or densities). Thus, each ρ^(*ij*)^ in eq 2 is represented through a distributed multipole expansion (DME)^51^ fitted for each *ij*, consisting of a partial charge, dipole moment, and quadrupole moment for each solute atom, which reproduce the electrostatic potential of the reference density. The interaction term *X*_*ij*_ can then be rewritten as
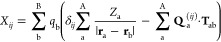
3where **Q** _a_^(*ij*)^ is the vector collecting the monopole, dipole, and quadrupole charges for atom a and state pair *ij* and **T**_ab_ is the corresponding geometric tensor of the multipole expansion.^51^ As discussed previously,^35^ the DME parameters **Q** _a_^(*ij*)^ depend on molecular orientation, so we use the Kabsch algorithm^52^ to transform the coordinates at each time step into the reference coordinate system. This allows computing rotationally invariant gradients and nonadiabatic couplings in LVC/MM.

## 3 Computational Details

The investigation of the coupled solute–solvent excited-state dynamics of CH_2_S and CMe_2_S consists of several steps that will be described in detail in the subsections below. In short: First, a set of LVC parameters (reference harmonic oscillator, vertical shifts, linear intrastate and interstate couplings, spin–orbit couplings, and DME parameters) was generated for each molecule. Second, we set up and pre-equilibrated both molecular systems (CH_2_S and CMe_2_S in water) using classical molecular dynamics in AMBER.^53^ Third, we performed LVC/MM simulations with SHARC^38,39^ in the electronic ground state to finalize the equilibration and produce initial conditions. Fourth, we carried out nonadiabatic LVC/MM TSH simulations after excitation to the S_1_ state. Afterward, the results of the simulations were analyzed. The analysis of the solvent distribution dynamics and the required number of trajectories—one of the main foci of this work—are described separately in Section 4.

### 3.1 LVC Setup

For both molecules, the S_0_ was optimized and frequencies were calculated with RIJCOSX-MP2^54^ and the cc-pVTZ basis set^55,56^ using the ORCA program package.^57^ These calculations provided the reference harmonic oscillators used in the LVC models (usually called *V*_0_ in the LVC literature^35^). The vibronic coupling parameters were evaluated using finite differences^37,58^ with multistate complete active space second-order perturbation theory (MS-CASPT2), using OpenMolcas v22.06.^59^

The CASPT2 calculations used the cc-pVTZ basis set,^55,56^ an IPEA shift of 0.25 au,^60^ an imaginary level shift of 0.1 au,^61^ and the Cholesky decomposition. For CH_2_S, we used an active space of 10 electrons in 9 orbitals, covering the sulfur lone pair, the π/π* pair, and the σ/σ* pairs of the three bonds (see Figure S2). The state-average complete active space self-consistent field (CASSCF) and MS-CASPT2^62^ computations included the first 9 singlet and first 9 triplet excited states, to stabilize the active space. For CMe_2_S, we used a smaller active space of 6 electrons in 5 orbitals (lone pair, π/π*, σ/σ* of the C=S bond, see Figure S3) as we encountered convergence issues with the (10,9) active space. This smaller active space was found to be stable when using the first 5 singlet and 4 triplet states. Based on previous work,^41^ different active spaces can be expected to only affect overall excitation energies but not excited-state energy gaps or geometries. For both molecules, the LVC models were constructed for the first four states (S_0_, S_1_, T_1_, and T_2_).

The DME parameters^63^ needed for electrostatic embedding were obtained by applying the restrained electrostatic potential (RESP) fit method^64^ (extended to treat multipoles^35^) to the relaxed electronic and transition densities. The extraction of these densities from OpenMolcas calculations for the subsequent RESP fits was newly implemented in the SHARC–OpenMolcas interface as part of the present work. In the RESP fits, we used the same settings as in our previous work,^35^ i.e., the original Merz–Singh–Kollman scheme and vdW radii, and a Lebedev quadrature with a density of 10 points/Å^2^. The used restraint parameter *c*_2_ (see eq 33 in ref (35)) was 0.0005, 0.0015, and 0.003 (in units of elementary charge) for monopoles, dipoles, and quadrupoles, respectively.

The LVC model parameter files for both molecules are provided in the Supporting Information, whose contents are summarized in Figure S4.

### 3.2 System Preparation

Both solute–solvent systems were prepared using tools from the AMBER package.^53^ The molecules were solvated in a 15 Å truncated octahedron box of TIP3P^65^ water (1091 and 1155 water molecules, respectively), as in our previous work.^35^ Using a 2 fs time step, periodic boundary conditions, SHAKE^66^ for bonds involving H, and GAFF2 parameters^67^ for the solutes, the systems were then relaxed for 1000 steps to remove bad contacts and cavities from the initially generated box, heated for 50 ps to 300 K (*NVT* ensemble), and equilibrated for 50 ns at 300 K and 1 bar (*NpT* ensemble). The final coordinates and velocities were reimaged into the original box (with the solute centered) and converted to the SHARC initial condition format.^68^

AMBER input files are given in the Supporting Information, whose contents are summarized in Figure S4.

### 3.3 SHARC Dynamics

As we showed previously,^35^ the first solvation shell of CH_2_S cannot be correctly simulated without including quadrupole charges on the S atom. Hence, the final snapshot of each AMBER trajectory was re-equilibrated using LVC/MM in the S_0_ to sample the initial conditions needed for TSH. The S_0_ simulations for both molecules were run with S_0_-only LVC-parameter files using a 2 fs time step. All solute and solvent distances involving H atoms were constrained to their equilibrium distances using the RATTLE algorithm implemented in SHARC 3.0.^69,70^ We simulated 1 ns employing a Langevin thermostat^71^ set to 293.15 K with a friction constant of 0.02 fs^–1^, a droplet potential,^72^ and a tether potential for the solute^35^ (for further details, see the input files provided in the Supporting Information). The first 50 ps serve as a re-equilibration step (to adapt the system from the force-field-based ensemble to the ensemble corresponding to LVC/MM) and were discarded. Snapshots were taken every 100 fs for the remaining 0.95 ns, producing 9500 and 9499 initial conditions for CH_2_S and CMe_2_S, respectively.^73^ This very large number of initial conditions is rationalized below in Section 4.2.

Using these sets of initial conditions for the two molecules, we performed TSH simulations including the entire water droplet. For comparison, we also performed TSH simulations in the gas phase by deleting all water molecules from the initial conditions. All initial conditions were propagated with a 0.5 fs time step for 3 ps after excitation to the S_1_. The TSH electronic wave function was propagated with a 0.02 fs time step using the local diabatization method.^74,75^ An energy-based decoherence correction scheme^76^ was applied to the electronic wave function, taking only the kinetic energy of the solute atoms into account. Likewise, the velocity vector rescaling after a hop was only applied to the solute atoms.

To reduce the produced TSH data to a manageable amount, data were stored every 5 fs during the first 100 fs and every 50 fs thereafter, for 79 data points per trajectory. For a subset of 500 trajectories, all time steps were retained for the analysis of the evolution of electronic populations. For reference purposes, we furthermore simulated trajectories in the S_0_ for 1 ps, starting from the same initial conditions but with their initial velocity vectors multiplied by −1.

SHARC input files and setup/analysis scripts are given in the Supporting Information, whose contents are summarized in Figure S4.

## 4 Details on Solvent Dynamics Analysis

In this section, we discuss the novel aspects of analyzing the three-dimensional solvent distribution and its dynamics around a photoexcited solute. We first discuss how the distribution functions should be computed and then discuss the number of trajectories required to achieve a certain spatial resolution at an acceptable noise level.

### 4.1 Trajectory Analysis

One of our primary goals is understanding the structure and dynamics of the solvation shell around the molecule. Hence, the nuclear coordinates from all the trajectories (totaling coordinates of about 4 billion atoms per system) were analyzed using two different kinds of distribution functions.

First, we compute radial distribution functions (RDFs) between the solute S atom and the water O atoms. The bin width was chosen as 0.05 Å and the RDFs were normalized by dividing with 4π*R*^2^ d*Rn*_traj_ρ_water_. The RDFs extracted from the S_0_ trajectories served as reference to calculate difference RDFs. The temporal evolution was then extracted using singular value decomposition (SVD) of the difference RDFs, ΔRDF(*R*, *t*) = ∑_*i*_*V*_*i*_(*R*)·*s*_*i*_·*U*_*i*_(*t*).

Second, as the RDFs cannot capture the complete solvent shell dynamics around the molecule, we also compute three-dimensional histograms of water O and H atom occurrences around the solute. We call these three-dimensional histograms “3D spatial distribution functions” (3D-SDFs) below. One important difference between the RDF and 3D-SDF analyses is that the RDFs are invariant under rotation of the solute (because they depend only on interatomic distances), whereas the 3D-SDFs do depend on the solute’s orientation. Thus, for the 3D-SDF analysis, the trajectory coordinates need to be aligned before aggregating the histograms. In the present work, this was performed with two different protocols. Both protocols can be defined in terms of a transformation operator  for every trajectory *k*, which is obtained as a function . This operator superimposes the nuclear coordinates of the solute **R**_*k*_^(solute)^(*t*) in trajectory *k* at time *t* onto the solute reference coordinates **R**_ref_^(solute)^. The operator is obtained through the Kabsch algorithm^52^ and applies a series of translation, rotation, and translation to a set of solute and/or solvent coordinates (details are given in Section 2.2 of ref (35)).

In the first of the two protocols, for each trajectory the solute coordinates **R**_*k*_(*t*) of all time steps are superimposed onto a common reference structure
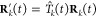
4

In this way, the solute serves as a fixed reference, allowing to aggregate the solvent distribution relative to the solute. Thus, we call the so-obtained 3D-SDFs to be from the “molecule’s perspective”. Because this perspective describes where the solvent is relative to the molecule (and its functional groups), it allows the direct interpretation of the solvent–solute interactions (e.g., solvation shifts of different electronic states). However, in this perspective, one can only observe the apparent motion of the solvent relative to the solute, whereas it is not possible to observe the actual solvent motion in an outside, fixed laboratory frame. Thus, intrinsic solvent relaxation timescales, diffusion coefficients, and similar quantities will be inaccessible.

In the second protocol, each trajectory is superimposed onto the reference using the transformation operator for *t* = 0 fs
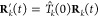
5In this way, the solute trajectories will diverge from the reference orientation, so it is not possible to investigate the solvent dynamics relative to the solute. However, this perspective features a fixed, inertial frame of reference, meaning one can observe the intrinsic solvent fluctuations in three-dimensional space, giving access to solvent relaxation timescales. Thus, this is the “solvent’s perspective”.

The 3D-SDFs were collected using the cpptraj program from AmberTools23.^53^ We employed three-dimensional grid cells with an edge length of 0.5 Å (i.e., with volume of 0.125 Å^3^). This value is approximately equivalent to the highest resolutions in experimental X-ray crystallography achieved to date^77,78^ and is also approximately equal to the radius of a H atom and hence corresponds roughly to the smallest features of atomic positions that are desirable to be resolved in the solvent distribution. All depictions of the solute molecules and the 3D-SDFs were composed using the Jmol program.^79^ The 3D-SDF temporal evolution was also analyzed using SVD, ΔSDF(*x*,*y*,*z*,*t*) = ∑_*i*_*V*_*i*_(*x*,*y*,*z*)·*s*_*i*_·*U*_*i*_(*t*).

### 4.2 Number of Trajectories

The last point to discuss here is the estimation of the number of trajectories required to resolve the solvent dynamics via the 3D-SDFs with a desired grid spacing. The expectation value of the number of O atoms (and thus water molecules) in a grid cell is given by
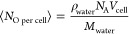
6where ρ_water_ is the density of water, *N*_A_ is Avogadro’s constant, *V*_cell_ is the volume of a grid cell, and *M*_water_ is the molar mass of water. At room temperature, this corresponds to about 0.0042 water molecules per grid cell (with *V*_cell_ = 0.125 Å^3^), showing that the grid cells are significantly smaller than the solvent molecules.

The standard deviation of ⟨*N*_O per cell_⟩ due to density fluctuations^80,81^ can be computed from
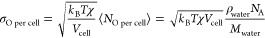
7where *k*_B_ is Boltzmann’s constant, *T* is the temperature, and χ is the isothermal compressibility (about 0.45 GPa^–1^ for water at standard conditions^82^). Comparison of eqs 6 and 7 shows that the number of water molecules per cell grows linearly with *V*_cell_ while the standard deviation grows with . Hence, larger grid cells will provide less noisy 3D-SDFs, although at the cost of reducing the spatial resolution of the investigated solvent dynamics. Thus, a more expedient way to reduce the noise of the 3D-SDFs is to employ several trajectories. This is equivalent to replacing *V*_cell_ by *n*_traj_*V*_cell_ in eqs 6 and 7, so that the final relative standard deviation is
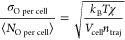
8

To adequately estimate the noise level of the 3D-SDFs, we also need to consider that for very small grid cells, as the ones used here, the effective isothermal compressibility is much higher than in the macroscopic limit.^81^ In Figure S5, we estimate the effective isothermal compressibility of TIP3P water to be 7.32 GPa^–1^ for a grid spacing of 0.5 Å. With this value, eq 8 becomes approximately .

**Figure 2:**
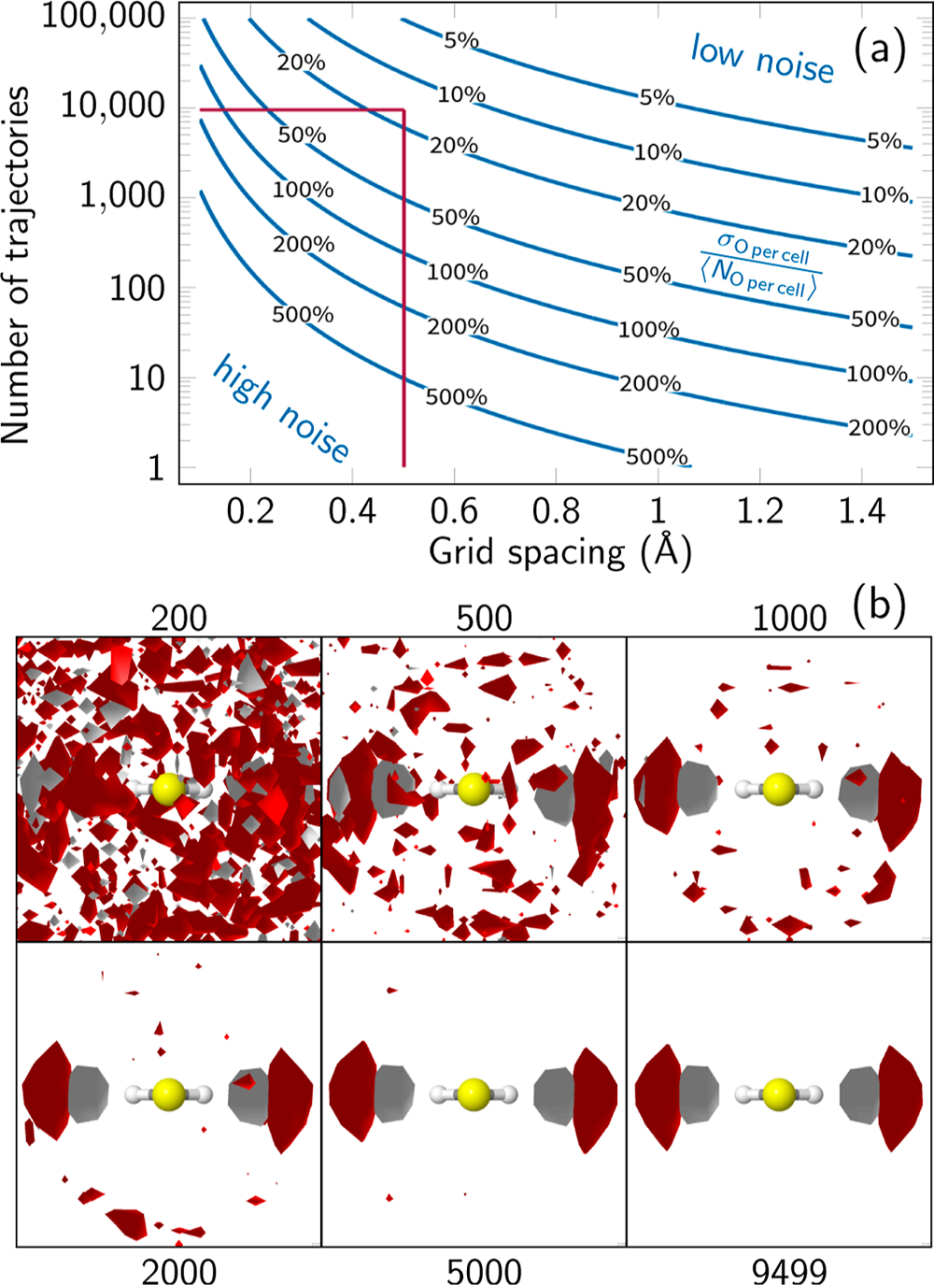
(a) Contour plot of  as a function of *V*_cell_ and *n*_traj_ for TIP3P at standard conditions and χ = 7.32 GPa^–1^ (eq 8) and (b) comparison of 3D-SDFs around CH_2_S (molecule’s perspective) for different numbers of trajectories. The red lines in (a) indicate the values for *V*_cell_ and *n*_traj_ chosen for the analysis in this paper. The 3D-SDFs in (b) were plotted at the same relative isovalue of 3*n*_traj_⟨*N*_O per cell_⟩ with red and gray indicating occurrences of oxygen and hydrogen atoms, respectively.

The relation in eq 8 is shown in the contour plot in Figure 2a, providing the relative standard deviation as a function of the grid spacing and *n*_traj_ with χ = 7.32 GPa^–1^. A trajectory number of 100 (typical for many TSH studies) thus would produce a relative standard deviation of 155% in the 3D-SDF, making the identification of significant solvent dynamics very difficult. Instead, 1000 trajectories correspond to a 50% deviation and 10,000 trajectories to 16% deviation. Figure 2b provides examples that showcase how well the features of the solvent distribution can be discerned in the 3D-SDFs for different numbers of trajectories at *t* = 0 fs. Figure S6 provides additional examples for 100 trajectories and for later times along the trajectories.

We note that the trajectory number estimation also in principle requires to know how strongly anisotropic the solvent distribution is around the solute and how much the distribution changes during the dynamics. Here, strongly increased local solvent density (e.g., from ground-state hydrogen bonds) will be easier to observe than weak features (e.g., the second solvation shell). Observing weaker features requires to lower the isovalue in the 3D-SDF plots, but this also will produce more noisy plots. To obtain sufficiently noise-free plots, the isovalue should be larger than the average number of atoms per cell, plus three standard deviations. This aspect is exemplified in Figures S7 and S8, which show the 3D-SDFs for CH_2_ at *t* = 0 fs and *t* = 100 fs for different number of trajectories and different isovalues. In Figure S7, the first column uses a relative isovalue of 5 (i.e., isovalue of 5*N*_traj_⟨*N*_O per cell_⟩). Thus, only the strongest features, the very strong ground-state hydrogen bond, are visible, and only 500 trajectories are needed to suppress the noise in the plot. Unfortunately, as Figure S7 (first column) shows, at a relative isovalue of 5 also the evolving solvent distribution at *t* = 100 fs is completely suppressed. A lower relative isovalue of 3 (Figures S7 and S8, second column) allows observing the photoinduced solvent dynamics, but full noise suppression requires at least 2000 trajectories. At relative isovalues of 1.66 and 2.0, the entire first solvation shell can be observed with ≥5000 trajectories. At an isovalue of 1.33, one can even observe a very shallow second solvation shell, although a huge number of trajectories (≥10,000 trajectories) is needed to resolve the solvation shell over the random fluctuations around the average density (Figures S7 and S8, last column) that takes place throughout the box and that is quantified by eq 8. However, we note that for the remainder of the discussion, we focus on the hydrogen bond regions, which are the most interesting aspect due to their interaction with the solute’s excited-state dynamics.

Overall, Figure 2 indicates that to observe the dynamics of rather strong hydrogen bonds, a trajectory number between 1000 and 10,000 seems to be sufficient. We assume that similar numbers are required for other solvents but would recommend that approximate effective isothermal compressibilities should be computed at the beginning of 3D-SDF analyses as described above.

## 5 Results and Discussion

In the following subsections, we first briefly recapitulate the excited-state electronic structure of CH_2_S and CMe_2_S. We then discuss the observed solvent relaxation dynamics by means of the RDFs, then by the 3D-SDFs, and finally the electronic dynamics, which is strongly coupled to the solvent dynamics.

### 5.1 Electronic Structure in the Gas Phase

The electronic structure of CH_2_S^41^ has four low-lying (<3.5 eV) electronic states, which are the closed-shell ground state (S_0_), the singlet ^1^nπ* state (S_1_), the triplet ^3^nπ* state (T_1_), and the triplet ^3^ππ* state (T_2_). The gas-phase vertical excitation energies are 2.26, 1.99, and 3.48 eV for the S_1_, T_1_, and T_2_ states, respectively (see Figure 1). Higher-lying excited states are experimentally measured and theoretically predicted to occur well above 5 eV.^43^ The electronic structure of CMe_2_S is analogous, with vertical excitation energies of 2.47, 2.30, and 3.35 eV for S_1_, T_1_, and T_2_, respectively (see Figure S1). It should be noted that the S_0_ and T_2_ states both have a high electron density in the molecular plane due to the doubly occupied S lone pair; in contrast, the S_1_ and T_1_ states (nπ*) have an increased electron density above and below the molecules. This results in differences in the electrostatic potential generated by the different states, where S_0_ and T_2_ have minima in the molecular plane and S_1_ and T_1_ perpendicular to it. These differences between the states can be recognized from Figures S9 and S10, which show the electronic state and transition densities and their exerted electrostatic potential.

The differences in electron densities are expected to drive the solvent relaxation dynamics. In the S_0_ and T_2_, hydrogen bonds to S are formed in the molecular plane.^35^ These hydrogen bonds stabilize S_0_ and T_2_, reducing the S_1_–T_2_ gap (compared to gas phase) and enhancing the possibility of ISC (see Figures 1 and S1). However, excitation to S_1_ is expected to lead to a redistribution of hydrogen bonds to out-of-plane positions, which will stabilize S_1_ and T_1_, widening the S_1_–T_2_ gap over time and shutting down ISC.

### 5.2 Time-Resolved Solvent Dynamics: RDFs

**Figure 3:**
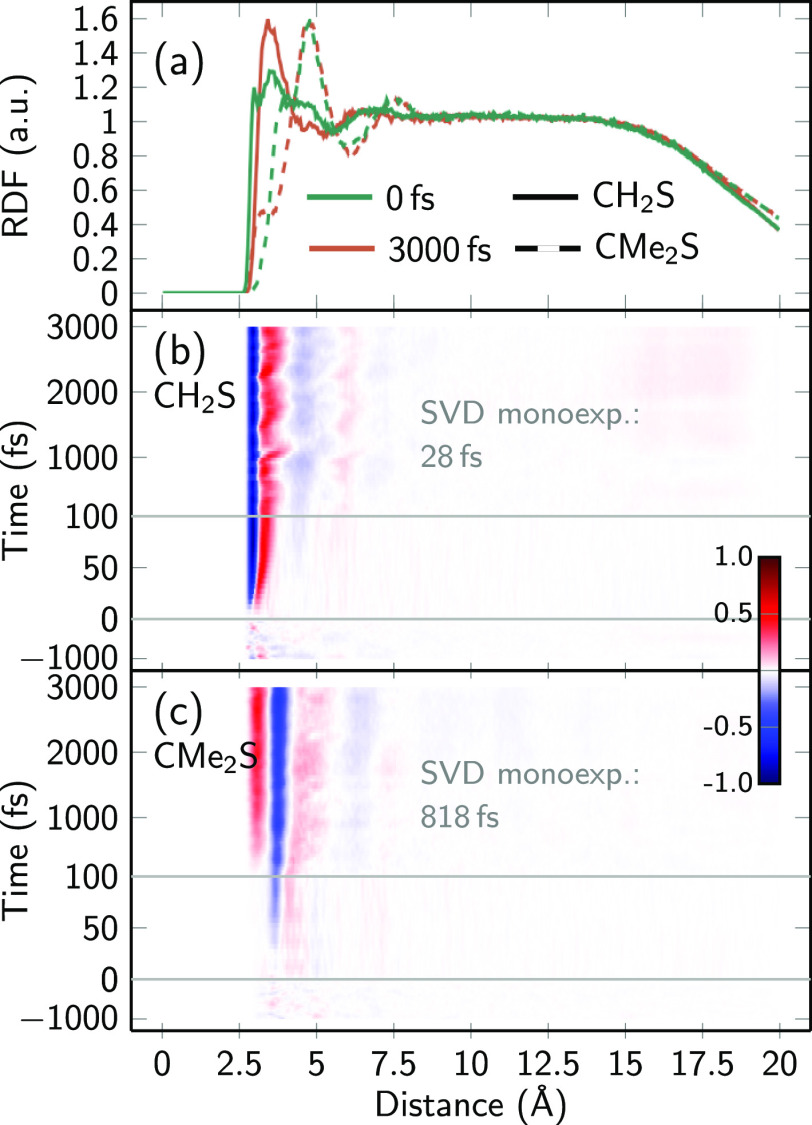
(a) RDFs for both molecules at times 0 and 3000 fs and (b,c) time-resolved difference RDFs between the S atom and water O atoms. For the difference RDFs, the reference is the average RDF between −1000 and 0 fs. Note the three-range split of the time axis in (b,c).

Figure 3a shows the average RDF between the S and water O atoms for both systems at *t* = 0 fs (green) and at *t* = 3000 fs (orange). The initial RDFs differ rather strongly between the molecules: CH_2_S exhibits a small maximum at 3.0 Å and a slightly stronger maximum at 3.5 Å. In contrast, CMe_2_S has a shoulder at 3.8 Å and a much higher maximum at 4.8 Å. After 3000 fs, the RDFs have changed slightly. In CH_2_S, the small 3.0 Å maximum vanishes and the main peak increases and slightly shifts to 3.4 Å, whereas in CMe_2_S the shoulder weakens and shifts to 3.1 Å but the maximum remains unchanged. We note that the RDFs show an extended flat region between 8 and 13 Å, which shows that the droplet is large enough to contain the structured solvation shell around the molecule and an additional 5 Å buffer region to the droplet surface. We assume that this is large enough to avoid significant perturbations of the solute–solvent dynamics by boundary effects, even though no periodic boundary conditions are used.

Figure 3b,c shows the temporal evolution of the difference RDFs, where the average RDF between −1000 and 0 fs serves as a reference. Note that we use difference RDFs instead of absolute RDFs for clarity, as is common in the X-ray scattering community.^83^ The difference RDFs for negative times show random fluctuations in the equilibrated ground state, exhibiting very low noise levels. Significant changes in the RDFs can be observed at positive times, after excitation of the solute. In the case of CH_2_S, the RDF around 2.9 Å decreases already after 20 fs and increases at 3.1 Å; for later times, this pattern simply grows further. This suggests that the water O atoms withdraw from the S atom as a result of the change in solvent distribution. An exponential fit to the first SVD component of the difference RDF yields a time constant of approximately 30 fs. This is consistent with the reported inertial response times for solvent relaxation dynamics in water,^8^ although the 3D-SDF analysis below will provide further insights into this solvent dynamics.

Interestingly, the increase–decrease pattern is different for CH_2_S than for CMe_2_S. For the latter, water actually approaches the solute after excitation, which can be recognized from the shift of the first shoulder toward a lower distance in Figure 3a. Figure 3c shows much slower dynamics for CMe_2_S. Here, after about 50 fs a small decrease around 3.7 Å appears, and after several 100 fs an increase of around 3.0 Å arises. The fit of the first SVD component gives a time constant of about 800 fs, more than 1 magnitude slower than for CH_2_S.

### 5.3 Time-Resolved Solvent Dynamics: 3D-SDFs

**Figure 4:**
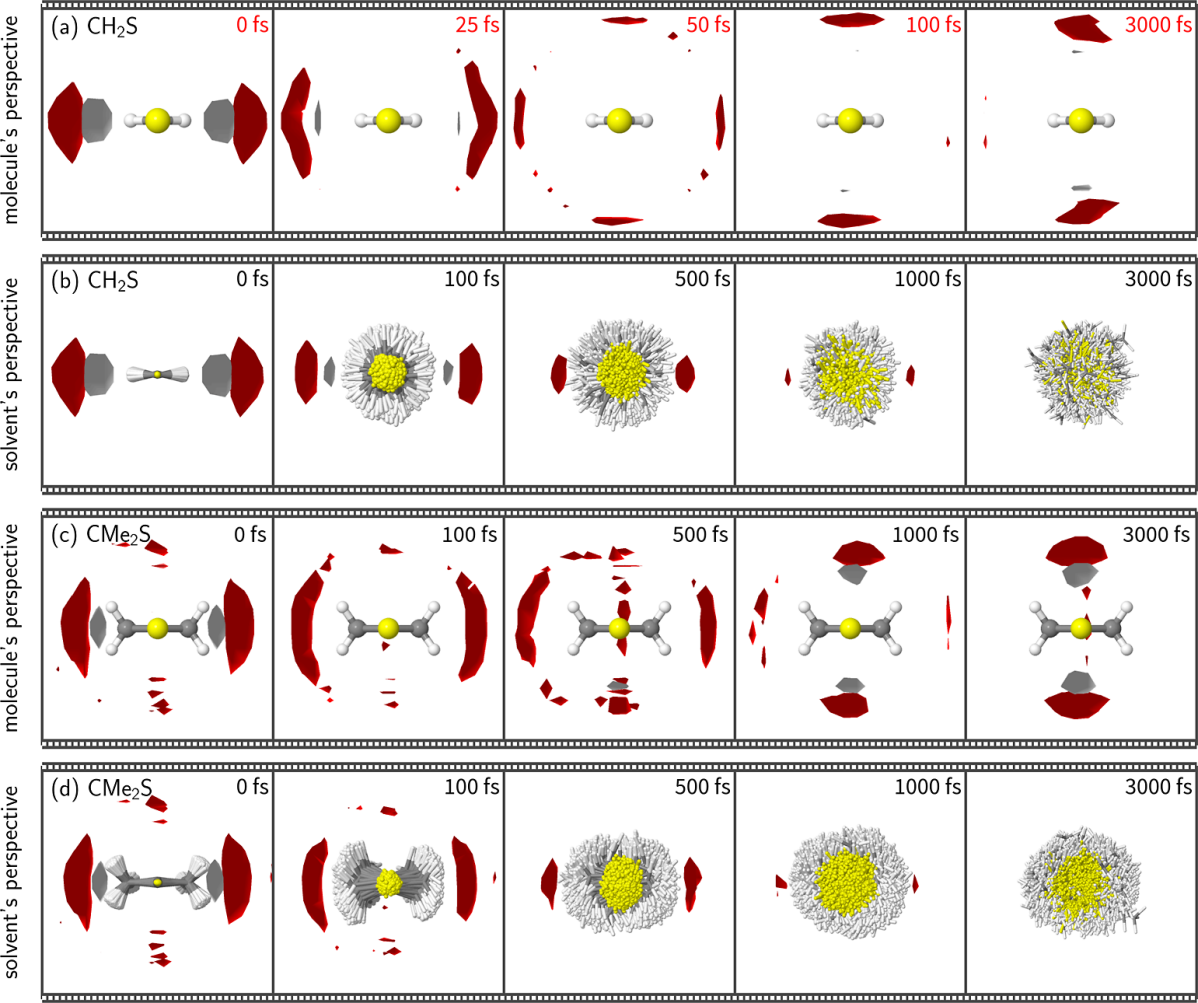
Time-resolved 3D-SDFs of water H and O atom occurrences around (a,b) CH_2_S and (c,d) CMe_2_S. The solvent dynamics is displayed in two different ways, from the (a,c) molecule’s perspective and (b,d) from the solvent’s perspective (see Section 4.1). In (a,c), all snapshots are aligned to the ground-state equilibrium of the respective solute molecule which is depicted. The gray and red regions indicate H and O occurrences with an isovalue of 3*N*_traj_⟨*N*_O per cell_⟩. Note that in (a) different points in time are shown than in the other panels because the solvent dynamics around CH_2_S in the molecule’s perspective has a much shorter timescale than the ones in the other panels, as discussed in the text.

The RDFs provide only a limited picture of the actual solvent reorganization dynamics around the molecule because they do not include any angular or spatial information. Therefore, in Figure 4 we show the obtained time-dependent 3D-SDFs of water around the solute molecules. Due to the very large number of trajectories (9500), the 3D-SDFs are almost free of distracting noise, even though we employ a resolution of 0.5 Å and do not employ any temporal averaging for maximum time resolution. As discussed in Section 4.1, we produced two sets of 3D-SDFs per molecule, from the “molecule’s perspective” and “solvent’s perspective”, which will be discussed step-by-step in the following.

At *t* = 0 fs, all four panels show very similar pictures of two strong in-plane hydrogen bonds of water to the solute S atom. The pictures agree with each other because by definition the two perspectives are identical at *t* = 0 and because the S_0_ electron density around the S atom is very similar for both molecules.

**Figure 5:**
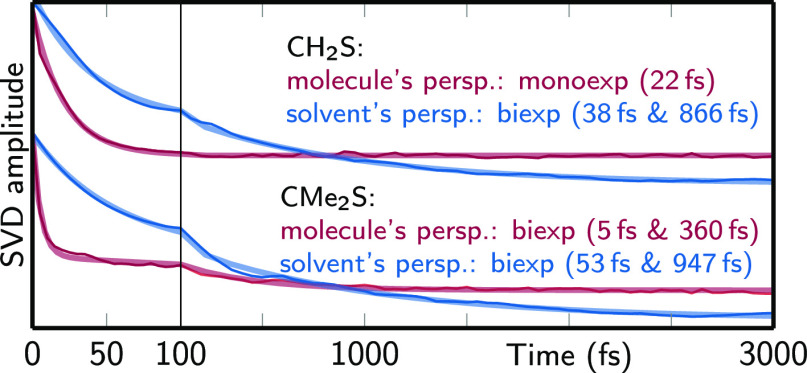
First temporal components *U*_1_(*t*) of SVDs of the 3D-SDFs shown in Figure 4a–d. The given time constants are obtained by mono- or biexponential fits of *U*_1_(*t*). Note that the first SVD component only captures part of the solvent dynamics and thus provides only a qualitative time constant to compare the two molecules.

Figure 4a employs the molecule’s perspective, where the 9500 geometries at each time step are aligned with the reference structure. Hence, in this perspective, the CH_2_ molecule stays fixed at the center of the images, and the solvent evolves around it. It can be seen that already 25 fs after excitation, the hydrogen bonds are broken and the water molecules start “flowing” around the molecule toward the out-of-plane positions. It is also visible that the H atom density drops faster (less visible gray-colored region) than the O atom density, due to the higher mobility of the water H atoms. Two (weaker) out-of-plane hydrogen bonds to the C atom form within about 100 fs. For longer times, these hydrogen bonds seem to be rather stable and appear unchanged after 3000 fs. This can also be observed nicely in the Supporting Information Movie. We performed an SVD of the time-dependent 3D-SDF; a monoexponential fit of the first temporal component provides a time constant of approximately 20 fs, as given in Figure 5. We note that for the 3D-SDFs, the first SVD component can only capture part of the solvent relaxation because in the 3D-SDFs time and position are correlated. Thus, the obtained time constant should only be regarded as a rough characterization of the solvent relaxation time. This is an astonishingly small time constant for the breaking and reforming of hydrogen bonds after photoexcitation of a solute. Experimental studies typically report values of 1 to 2 magnitudes slower for such processes.^8,11,84,85^

In order to understand how the solvent relaxation dynamics around CH_2_S can be orders of magnitude faster than in other systems, we turn to Figure 4b, showing the 3D-SDF from the solvent’s perspective. Unlike the molecule’s perspective, the solvent’s perspective exhibits a fixed frame of reference, allowing us to discern the intrinsic flux of the solvent distribution through three-dimensional space. In Figure 4b (note the different time points plotted compared to panel a), we can observe that the water molecules that formed the two in-plane hydrogen bonds to the S atom are still approximately in the same position after 100 fs. This shows that the solvent distribution requires time to break the existing hydrogen bonding networks and form new hydrogen bonds in response to the excitation of the solute. The broad distribution of the CH_2_S molecule, which is rotated around its C–S bond, suggests that the molecular orientation changed drastically. At later times, one can see that the regions of high water occupancy gradually decay, being slightly visible at 1000 fs but vanished before 3000 fs. At the same time, the CH_2_S molecules continue to randomize their orientation due to diffusion processes.

In total, we find that CH_2_S simply rotates around the C–S bond to reform the hydrogen bonds and accommodate the new electronic state within the solvation shell in the fastest possible way, much faster than the intrinsic solvent rearrangement alone would allow. The timescale, extent, and final distribution of this rotation is shown in Figure S11a. In particular, the distribution shows a fast divergence within the first 100 fs and a slight peak after 100 fs at about 90°, which is the ideal angle to facilitate the excited-state out-of-plane hydrogen bonds. Figure S11a also shows large differences between negative times (in S_0_) and positive times (after excitation), showing that solute rotation in the excited state is not diffusion-driven. We characterized the solvent dynamics in the solvent’s perspective through an SVD of the 3D-SDF and fitted time constants of about 40 and 900 fs with a biexponential function (Figure 5a) Here, the first time constant corresponds to inertial/librational motion (i.e., hydrogen bond breaking), while the second one is in line with previous experimental measurements of water relaxation timescales.^8,11,84,85^ We note that in the solvent’s perspective, the fitted time constants only correspond to the decay of the initial solvent structure but do not describe the reformation of hydrogen bonds that was visible in the molecule’s perspective.

Compared to CH_2_S, the solvent relaxation dynamics of CMe_2_S shows some interesting differences. First, in Figure 4c it can be seen that, from the molecule’s perspective, the solvent shows the dissolution of the two in-plane hydrogen bonds to the S atom and the creation of two out-of-plane hydrogen bonds to the C atom, similar to CH_2_S. However, this process takes significantly longer in CMe_2_S than in CH_2_S, as visible by comparing panels a and c, e.g., panel a at 25 fs presents a similar solvent distribution as panel c at 100 fs, and panel a at 100 fs is comparable to panel c at 1000 fs. The much slower solvent dynamics in CMe_2_S can be explained in the solvent’s perspective in panel d. Here, it can be discerned that this molecule is strongly hindered in its rotation around the C–S bond due to the steric bulk (i.e., the drag) of the methyl groups. Hence, in CMe_2_S the reformation of the hydrogen bond network is limited by the timescale of the solvent rearrangement, unlike in CH_2_S where molecular rotation contributes to the reformation. The rotational dynamics of CMe_2_S is presented in Figure S11b, which shows that the rotation is effectively suppressed by the solvent cage. Interestingly, the time constants for the solvent relaxation in the solvent’s perspective (Figure 5) of CMe_2_S are about 50 and 900 fs, which agree very well with the ones of CH_2_S. This is in strong contrast to the time constants obtained from the molecule’s perspective (CH_2_S: 22 fs, CMe_2_S: 5 and 360 fs).

The time-dependent 3D-SDFs (Figure 4) clearly show more details of the solvent dynamics than the RDFs (Figure 3). Using the 3D-SDFs, it is even possible to explain why the difference RDFs show the withdrawal of water from excited CH_2_S but an approaching of water to excited CMe_2_S. The reasons are steric hindrance and how the excited-state hydrogen bonds are formed. In CH_2_S, the hydrogen bonds in the ground state are not sterically hindered and thus are very close to the S atom. In the excited state, the hydrogen bonds are more strongly attracted to the C atom, so they move further away from the S atom. An analysis of the hydrogen bond count over time (see Figure S12a) and a corresponding fit shows that the hydrogen bonds break within about 20 fs and reform within 200 fs in CH_2_S. Contrarily, in CMe_2_S, the hydrogen bonds in the ground state are slightly hindered by the methyl groups and thus are longer than in CH_2_S. In excited CMe_2_S, the out-of-plane hydrogen bonds are less obstructed, so they can approach the molecule more closely than in the ground state, leading to a shift of the RDF maximum to shorter values. Here, the hydrogen bonds break within 40 fs and reform after about 900 fs (see Figure S12b), significantly slower than in CH_2_S. We note that these time constants of hydrogen bond breaking/formation depend on the criteria for counting the hydrogen bonds and thus are only qualitative.

Although the RDFs and 3D-SDFs in principle describe the same dynamics, we note that the obtained time constants are not fully consistent. The time constants derived from the SVD of the difference RDFs (CH_2_S: 28 fs, CMe_2_S: 818 fs) are neither consistent with the time constants from the molecule’s perspective nor with the ones from the solvent’s perspective. This is remarkable, as both the RDFs and the solvent’s perspective 3D-SDFs describe the local solvent dynamics relative to the solute. However, the highly anisotropic dynamics visible in the 3D-SDFs around the thiocarbonyl groups simply cannot be represented in full detail in the difference RDFs.

### 5.4 Electronic Dynamics

**Figure 6:**
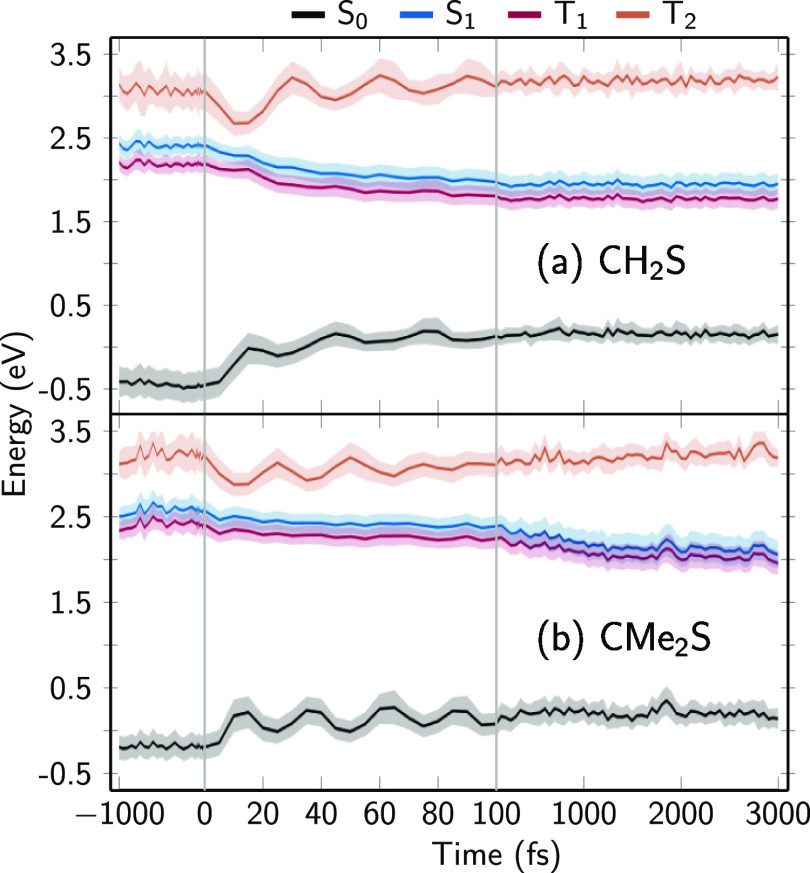
Eigenenergies of the electrostatically embedded LVC Hamiltonian matrix (excluding MM contributions) of the (a) CH_2_S and (b) CMe_2_S systems. The energies of S_0_, S_1_, T_1_, and T_2_ are shown in black, blue, red, and orange, respectively. Lines show the average over all trajectories and the shaded area indicates one standard deviation.

In addition to information about the solute’s and solvent’s nuclear degrees of freedom, the LVC/MM-TSH trajectories provide information about the electronic wave functions, analogous to ab initio TSH trajectories. Figure 6 shows the average eigenenergies of the coupled LVC Hamiltonian (eq 1) of the whole trajectory swarms; the shaded areas indicate one standard deviation. The figure shows that, for both molecules, the eigenenergies are approximately constant at negative times, indicating proper equilibration in the ground state. After excitation to the S_1_ state at *t* = 0 fs, one can observe strong oscillations in the S_0_ and T_2_ energies within the first 100 fs. These oscillations have periods of about 30 fs for CH_2_S and 25 fs for CMe_2_S, which correspond to about 1111 and 1333 cm^–1^, respectively. Both frequencies match the C–S stretching modes (frequencies of 1106 and 1336 cm^–1^, respectively), which are strongly excited in the S_1_ state (Figure S13).^41^

The C–S stretch mode strongly modulates the S_1_–T_2_ energy gap and thus mediates ISC. ISC is not possible in the gas phase because the S_1_–T_2_ energy gap never gets sufficiently small (Figures 1c and S1), as discussed in ref (41). In aqueous solution, the T_2_ state is stabilized by the hydrogen bonds formed in the S_0_. Thus, after 12 fs the average S_1_–T_2_ energy gap reaches a minimum. However, as shown in Figures 6 and S14, after excitation to S_1_ the S_1_ and T_1_ states eventually become stabilized by the solvent relaxation dynamics. In CH_2_S, the S_1_ is shifted by −0.4 eV within 100 fs, while in CMe_2_S, the S_1_ is shifted by a similar amount within approximately 1500 fs (see Table S1 for numerical values). These solvent-induced shifts widen the S_1_–T_2_ energy gap so much that ISC shuts down very quickly.

**Figure 7:**
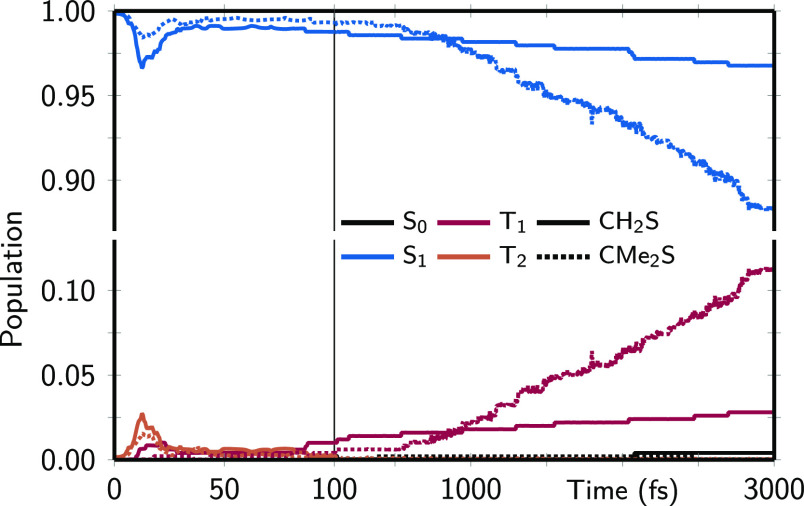
Evolution of electronic populations (spin-free states summed over triplet M_S_ components) for S_0_, S_1_, T_1_, and T_2_ in black, blue, red, and orange, respectively, of CH_2_S (solid) and CMe_2_S (dotted).

The population dynamics presented in Figure 7 is fully consistent with the dynamic singlet–triplet energy gap (see Figure S14) and with a time-dependent, very subtle mixing of the two triplet states (Figure S15). First, there is a rapid but small increase in the triplet population within the first 30 fs for both systems due to the closing of the S_1_–T_2_ energy gap (Figure 6). Here, ISC is more pronounced for CH_2_S because its singlet–triplet energy gap is slightly smaller. However, further transfer of population to the T_2_ is inhibited at later times because the solvent dynamics stabilizes the S_1_ and thus widens the S_1_–T_2_ energy gap. Trajectories in the T_2_ eventually return to the S_1_, as the T_2_ electron density favors small S_1_–T_2_ energy gaps, just like the S_0_ density. Figure S14b,c nicely shows the small amount of trajectories for which the S_1_–T_2_ energy gaps remain close to zero for about 100 fs.

At later times, instead one can observe direct and slow population transfer from S_1_ to T_1_. Based on the electronic characters of these states at the Franck–Condon point (both are nπ* states) and the El-Sayed rule,^86^ this is surprising; the spin–orbit couplings between the S_1_ and T_1_ are exactly zero in the diabatic basis of the LVC model. Figure S14 shows that the S_1_ → T_1_ ISC is enabled by nuclear motion that breaks the molecular symmetry so that the T_1_ and T_2_ states mix (Figure S15), giving rise to nonzero spin–orbit couplings between S_1_ and T_1_. For CH_2_S, this mixing can only occur by interaction of the solvent with the T_1_–T_2_ (or S_0_–S_1_) transition density, as all interstate coupling constants (λ parameters^37,58^) are zero by symmetry. On the contrary, for CMe_2_S there are four normal modes that have the correct symmetry to couple T_1_ with T_2_ (or S_0_ with S_1_), in addition to solvent interaction with the transition densities. The presence of intramolecular degrees of freedom that couple S_1_ and T_1_ by mixing is the reason why S_1_ → T_1_ ISC occurs significantly faster in CMe_2_S than in CH_2_, as visible in Figure 7. A second driving factor for faster ISC in CMe_2_S is the smaller S_1_–T_1_ energy gaps (Figure S14). A simple linear fit of the T_1_ population between 100 and 3000 fs yields a time constant of 26 ± 3 ps for CMe_2_S and 130 ± 40 ps for CH_2_S.

Overall, these results evidence the complex relationship between the solute’s electronic and the solvent’s nuclear degrees of freedom. The electron density of the ground state determines the initial solvent distribution, which in turn governs the solute’s excited-state energy gaps. The solute’s intramolecular motion drives ISC after excitation to S_1_, but ISC is influenced by the solvent in two ways. On the one hand, the solvent distribution adapts quickly to the S_1_ electron density—which shuts down sub-ps ISC to T_2_—while on the other hand, random solvent fluctuations perturb the solute’s symmetry and thus enable slow ISC to T_1_. Due to the similar electronic character, electrostatic potential, and potential energy surface, the ISC from S_1_ to T_1_ does not further affect the solute’s or solvent’s nuclear motion.

## 6 Computational Cost

We have already discussed the computational cost and scaling of the LVC/MM method in our previous work,^35^ although focusing on CH_2_S and the ground state. We found that LVC/MM is about 1 order of magnitude faster, compared to a QM/MM reference trajectory at the BP86/def2-SVP level of theory. The present work gives us the opportunity to further highlight the computational efficiency of LVC/MM in the context of excited-state simulations. All timings are given as averages and using a single core of an Intel Xeon E5-2650 v3 processor (2.30 GHz clock speed), unless otherwise stated.

As written above, both trajectory swarms (each with about 9500 trajectories and 6000 time steps) required a combined 114 million single-point calculations. For CH_2_S (1091 waters), each LVC/MM time step took 120 ms, each trajectory took 12.5 min, and the entire trajectory swarm thus cost about 2000 CPU h. For the slightly larger CMe_2_S (1155 waters), a time step took 400 ms, each trajectory took 40 min, and the entire swarm cost 7000 CPU h. This very modest computational cost can be compared to the cost of equivalent ab initio QM/MM trajectories. We computed one QM/MM trajectory for each system using the TDA-BP86/def2-SVP level of theory, as in our previous work.^35^ The single-core computational cost was found to be 25 h per trajectory (15 s/step) for CH_2_S and 102 h per trajectory (60 s/step) for CMe_2_S. For the entire trajectory swarms, the cost would therefore amount to about 240,000 CPU h and nearly 1,000,000 CPU h, respectively, for CH_2_S and CMe_2_S, which is feasible but requires significant resources. Thus, even compared to a very affordable excited-state electronic structure method like TDA-BP86, LVC/MM provides a speed-up of about 2 orders of magnitude.

However, the LVC models used in the simulations above were not parametrized from TDA-BP86 but rather from MS-CASPT2. Here, we simply estimated the expected cost from a gas-phase single-point calculation including gradients,^87^ without including the MM point charges or their gradients. Thus, the following estimates are lower bounds. For CH_2_S, an MS-CASPT2 single-point calculation took 6 min, which can be extrapolated to 600 h per trajectory and about 6 million CPU h for the swarm. For CMe_2_S, the cost is approximately ten times as large (i.e., 6000 h per trajectory, nearly as much as the entire LVC/MM swarm), yielding a staggering overall cost of about 57 million CPU h. This is clearly beyond the capabilities of many, if not most, current research groups. In contrast, LVC/MM is clearly affordable and exhibits a speed-up of 3 to 4 orders of magnitude over MS-CASPT2.

We also want to comment briefly on the amount of data produced. At every time step, SHARC^39^ stores information on the electronic states and wave function as well as coordinates and velocities of all nuclei. For systems with MM solvent, the latter data grow very quickly for long simulation time and large trajectory swarms. For either of the two systems (3277 atoms for CH_2_S, 3495 atoms for CMe_2_S), each time step produces about 160 kB of nuclear data. Thus, a trajectory with 6000 time steps corresponds to about 1 GB of data, and one trajectory swarm to about 9 TB. In order to reduce the amount of data, we have saved coordinate data only every 5 fs during the first 100 fs and every 50 fs thereafter, which requires about 120 GB per trajectory swarm. A higher time resolution in the 3D-SDFs without excessive memory usage can be achieved by dividing the trajectory swarm into batches, aggregating partial 3D-SDFs and then deleting the raw coordinate data of the current batch before continuing to the next batch.

## 7 Conclusions

In the present work, we have demonstrated the capabilities of the recently published LVC/MM method,^35^ which implements electrostatic embedding of linear vibronic coupling models in MM environments, for use in TSH. This extremely efficient method allows simulating tens of thousands of TSH trajectories for many picoseconds and including thousands of MM solvent molecules. In particular, we were able to simulate 9500 trajectories of 3 ps length in about 2000 CPUh for the smallest employed system. Therefore, the method makes it possible to sample the time-dependent 3D-SDFs around an excited solute. Such 3D-SDFs provide much more insights into solvent relaxation than the typically employed RDFs. However, reaching acceptable noise levels in the 3D-SDFs requires extensive sampling—several thousand trajectories for few-femtosecond and sub-Ångstrom resolution in water. We show that 3D-SDFs are affected by the solute’s orientation and rotation and therefore can be analyzed in different ways. To that end, we discuss the “molecule’s perspective” and the “solvent’s perspective”. The former employs a corotating coordinate system to map the time-dependent distribution of the solvent relative to the solute—this perspective is beneficial to interpret solute-focused spectroscopic observations governed by solute–solvent interactions. Conversely, the solvent’s perspective uses a fixed coordinate system to analyze the timescales of the intrinsic solvent reorganization in three-dimensional space.

The LVC/MM TSH method has been applied to two model thiocarbonyl compounds, thioformaldehyde (CH_2_S) and thioacetone (CMe_2_S). These molecules exhibit two in-plane hydrogen bonds to the S atom in the ground state. After excitation to the S_1_ state, the solvent-induced small singlet–triplet gap enables nonzero ISC yield, in contrast to the gas phase, where no ISC is observed. However, quickly after excitation, the solvent responds to the change in solute electron density, breaking the in-plane hydrogen bonds and forming two new out-of-plane hydrogen bonds to the C atom. This process widens the singlet–triplet gap, shutting down ISC. The relaxation timescales of the solvent depend strongly on the investigated solute. In CH_2_S, hydrogen bonds are broken and reformed via a fast and directed rotation around the C–S bond within only 100 fs. In CMe_2_S, rotation is sterically hindered by the methyl groups, so that hydrogen bond reformation takes approximately 1 ps. We expect that similar hydrogen bond dynamics is at play in larger (thio-)carbonyl compounds in aqueous solution, for example in the ultrafast ISC of thionated nucleobases.

## Supporting Information Available

Gas-phase and solvated energies for CMe_2_S, active space orbitals for both thiocarbonyls, explanations for supplementary input files, computed mean and standard deviation of 3D-SDF of TIP3P water box, noise level of 3D-SDFs using different numbers of trajectories and isovalues, electronic densities and electrostatic potentials around sulfur atoms of both molecules, time evolution of molecular rotation angle, hydrogen bond count analysis, shifted eigenenergies over time, convolutions of coherently excited normal modes over time, time-dependent spin–orbit couplings and energy gaps, and time-dependent overlap between the triplet states (PDF)
LVC/MM parameter files for both molecules, SHARC inputs for all solvated trajectories, scripts for system preparation, and scripts used for solvation analysis (ZIP)
Movie of the time evolution of the 3D-SDF in the molecule’s and solvent’s perspective for CH_2_S and CMe_2_S (MP4)
