# Supplementary material for: Resolving Photoinduced Femtosecond Three-Dimensional Solute–Solvent Dynamics through Surface Hopping Simulations
Source: J Chem Theory Comput. 2024 May 20;20(11):4738–50. doi: 10.1021/acs.jctc.4c00169 (PMC11171268; doi:10.1021/acs.jctc.4c00169)
Supplement: Supplementary file 1 — ct4c00169_si_001.pdf [file ct4c00169_si_001.pdf]

Supporting Information: Resolving Photoinduced Femtosecond Three-Dimensional Solute–Solvent Dynamics through Surface Hopping Simulations

Severin Polonius,[†,‡] David Lehrner,[†] Leticia González,[†,¶] and Sebastian Mai[*,†]

†*Institute of Theoretical Chemistry, Faculty of Chemistry, University of Vienna, Währinger Str. 17, 1090 Vienna, Austria.*

‡*University of Vienna, Vienna Doctoral School in Chemistry (DoSChem), Währinger Str. 42, 1090 Vienna, Austria.*

¶*Vienna Research Platform Accelerating Photoreaction Discovery, University of Vienna, Währinger Straße 17, 1090 Vienna, Austria.*

E-mail: sebastian.mai@univie.ac.at

Contents

S1 Energies in gas phase and solution **S-3**

S2 Orbitals **S-4**

S3 Contents of the electronic supporting information **S-5**

S4 Solvation dynamics analysis **S-6**

S5 Electrostatic potentials **S-10**

S6 Analysis of rotation around C-S axis **S-12**

S7 Temporal evolution of hydrogen bonds **S-13**

S8 Shifts in eigenenergies of the coupled LVC Hamiltonian **S-14**

S9 Coherent Excitation of Normal Modes **S-15**

S10 Time-Dependent Couplings **S-16**

S1 Energies in gas phase and solution

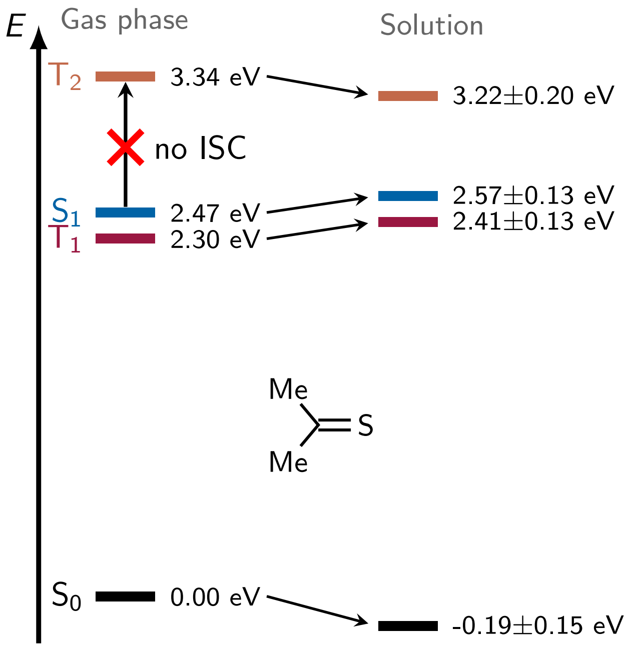

Figure S1: Jabłonski scheme of the gas phase and solvated ground state energies of CMe_2S (a scheme for CH_2S is shown in the main text) with MS-CASPT2(6,5) and the cc-pVTZ basis set.

S2 Orbitals

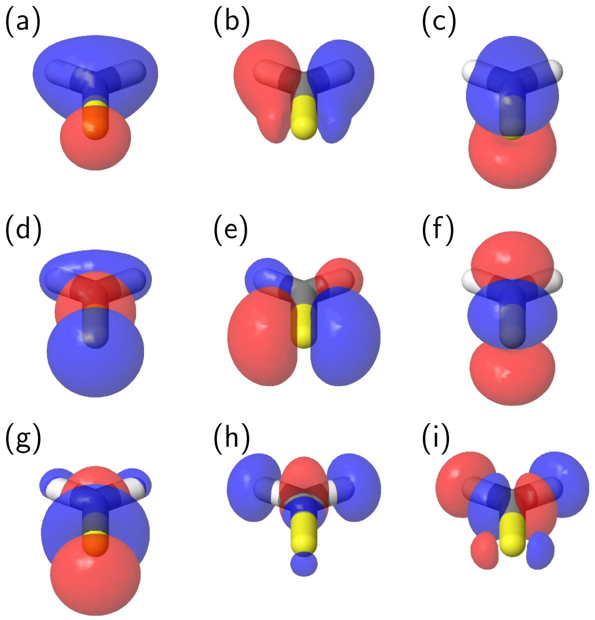

Figure S2: Active space orbitals used for the CASPT2 calculations on CH_2S (a: $\sigma_{\mathrm{CH}+}$ b: $\sigma_{\mathrm{CH}-}$ c: π d: σ_{CS} e: n f: π^* g: σ^*_{CS} h: $\sigma^*_{\mathrm{CH}+}$ i: $\sigma^*_{\mathrm{CH}-}$).

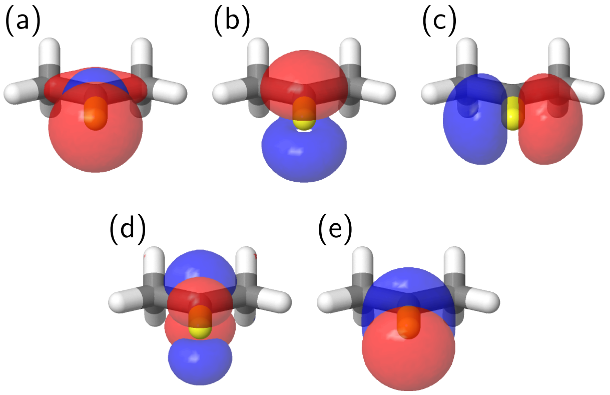

Figure S3: Active space orbitals used for the CASPT2 calculations on CMe_2S (a: σ_{CS}, b: π, c: n, d: π^*, e: σ^*_{CS}).

S3 Contents of the electronic supporting information

```
eSI/
├── CH2S/
│   ├── ch2s.prmtop
│   ├── LVC.template
│   ├── system.prmtop
│   └── V0.txt
├── CMe2S/
│   ├── cme2s.prmtop
│   ├── LVC.template
│   ├── system.prmtop
│   └── V0.txt
├── input_dynamics/
│   ├── sharc_input_S0_1ns_CH2S
│   ├── sharc_input_S0_1ns_CMe2S
│   ├── sharc_input_S1_3ps_CH2S
│   └── sharc_input_S1_3ps_CMe2S
├── scripts_for_system_preparation/
│   ├── run_Amber_min_heat_equ_prod.sh
│   └── run_cpptraj_reimage.sh
└── solvation_analysis_scripts
    ├── cpptraj_3d_hist_rdf.sh
    └── gather_traj_coords_netcdf_align_into_timeframes.py
```

Figure S4: Contents and file structure of the `.zip` file that accompanies this SI.
The folders `CH2S` and `CMe2S` contain the respective `LVC.template` and `V0.txt` files, as well as both `.prmtop` files used for the subtractive scheme in the LVC/MM calculations; `system.prmtop` contains the solute and solvent parameters and `<molecule>.prmtop` contains the solute parameters (solute charges are set to 0 in both files).
The folder `input_dynamics` contains the SHARC input files for the 1 ns long LVC/MM equilibration runs (in S_0) and the SHARC input files for the nonadiabatic dynamics simulations (from S_1) for an exemplary trajectory.
The folder `scripts_for_system_preparation` contains the scripts used to prepare the water boxes of both systems with AMBER and the script to subsequently reimage the coordinates.
The folder `solvation_analysis_scripts` includes two scripts,. The first one is `gather_traj_coords_netcdf_align_into_timeframes.py`, which extracts the coordinates from all trajectories, aligns the solute to the first time step and writes the aggregated coordinates into different files for each recorded time step. The second script is `cpptraj_3d_hist_rdf.sh`, which calculates the three-dimensional histograms of water oxygen and hydrogen atoms for molecule's and solvent's perspectives and the radial distribution functions between sulfur and water oxygen atoms.

S4 Solvation dynamics analysis

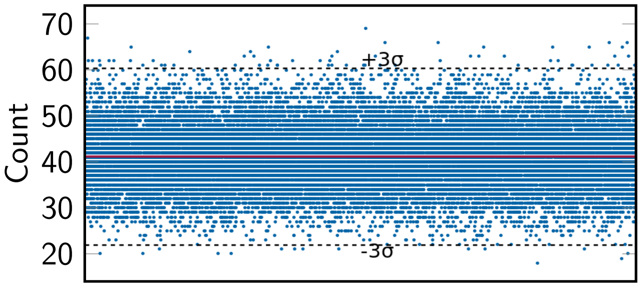

Figure S5: 3D histograms counts of TIP3P water box with grid size 0.5 Å of 10,000 snapshots. The simulations were carried out with AMBER with the same settings as described in Section 3.2, just without the solute molecule. The mean and standard deviation are $\langle N_{\mathrm{O\,per\,cell}} \rangle = 41.10$ and $\sigma_{\mathrm{O\,per\,cell}} = 6.40$, respectively (lines indicate μ (red) and $\langle N_{\mathrm{O\,per\,cell}} \rangle \pm 3\sigma_{\mathrm{O\,per\,cell}}$ (black)). Using Equation (8), the effective isothermal compressibility of TIP3P for the used grid cells is $7.32\,\mathrm{GPa}^{-1}$.

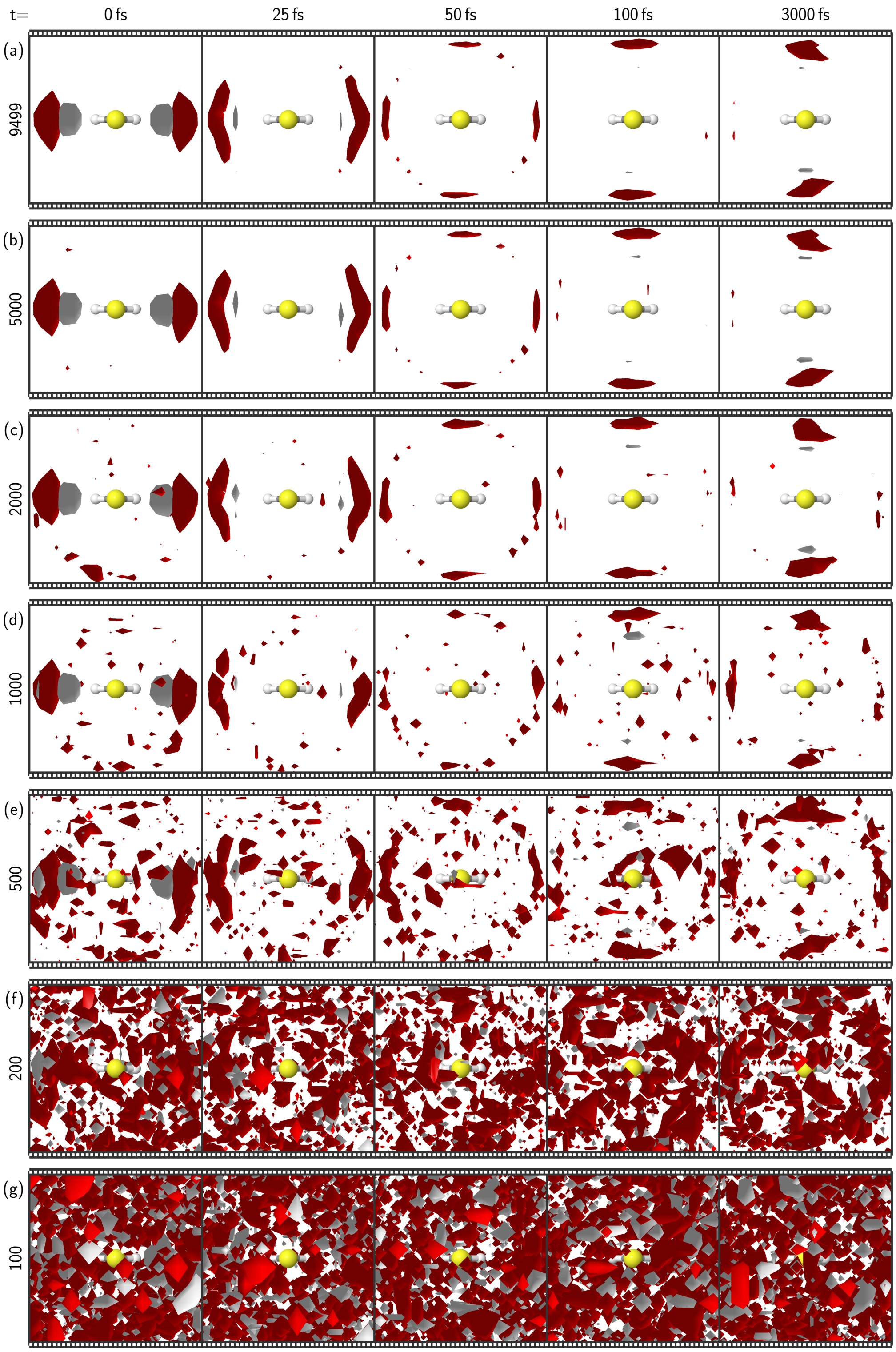

Figure S6: Comparison of time-dependent 3D-SDFs around CH_2S at different times after excitation from the molecule's perspective for different numbers of trajectories. The different points in time are given at the top, and the number of trajectories on the left of each panel.

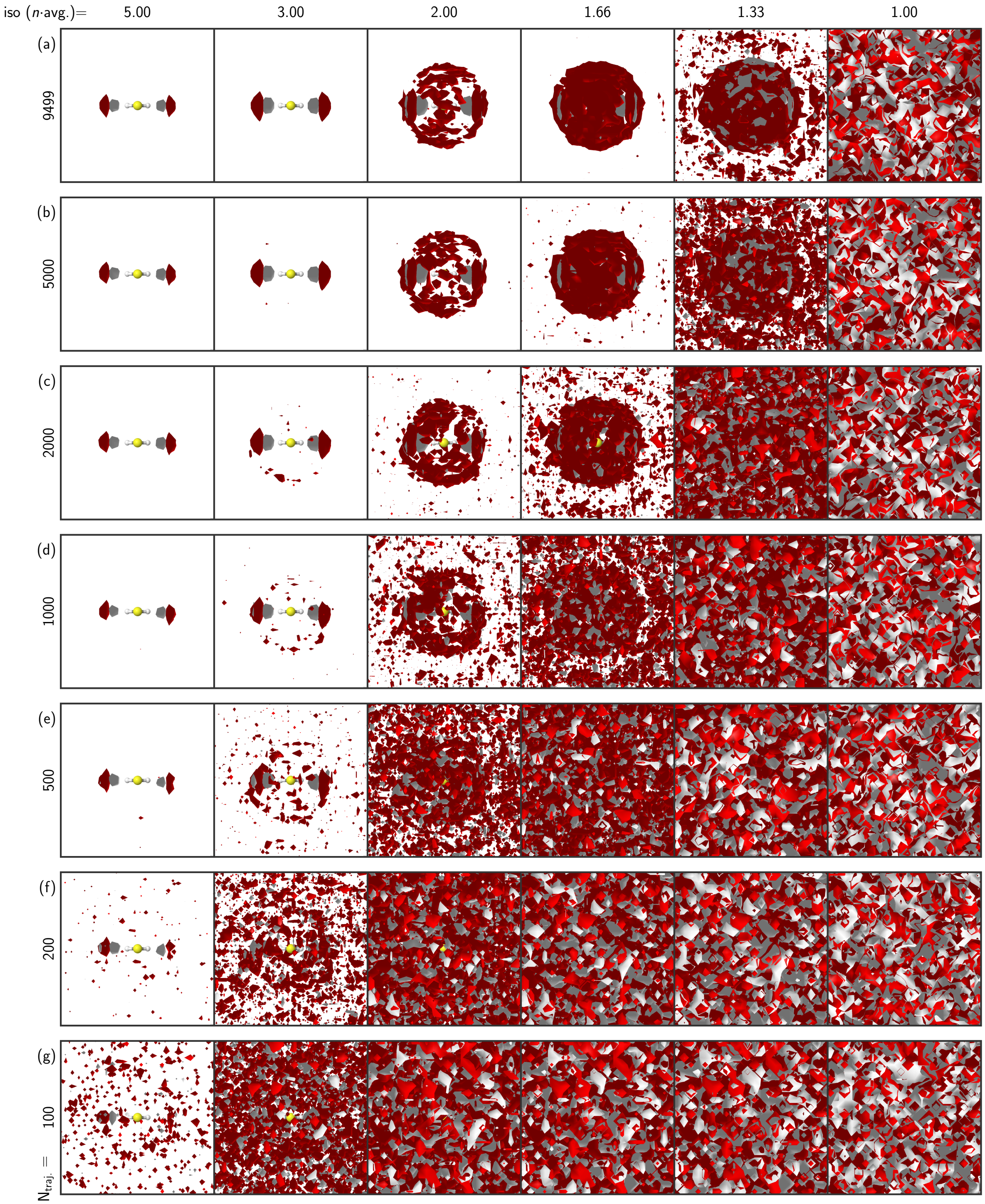

Figure S7: Comparison of time-dependent 3D-SDFs around CH_2S at $t = 0\,\mathrm{fs}$ from the molecule's perspective for different isovalues for different numbers of trajectories. The different isovalues are given in multiples of the average number of water oxygen and hydrogen atoms, and the number of trajectories on the left of each panel.

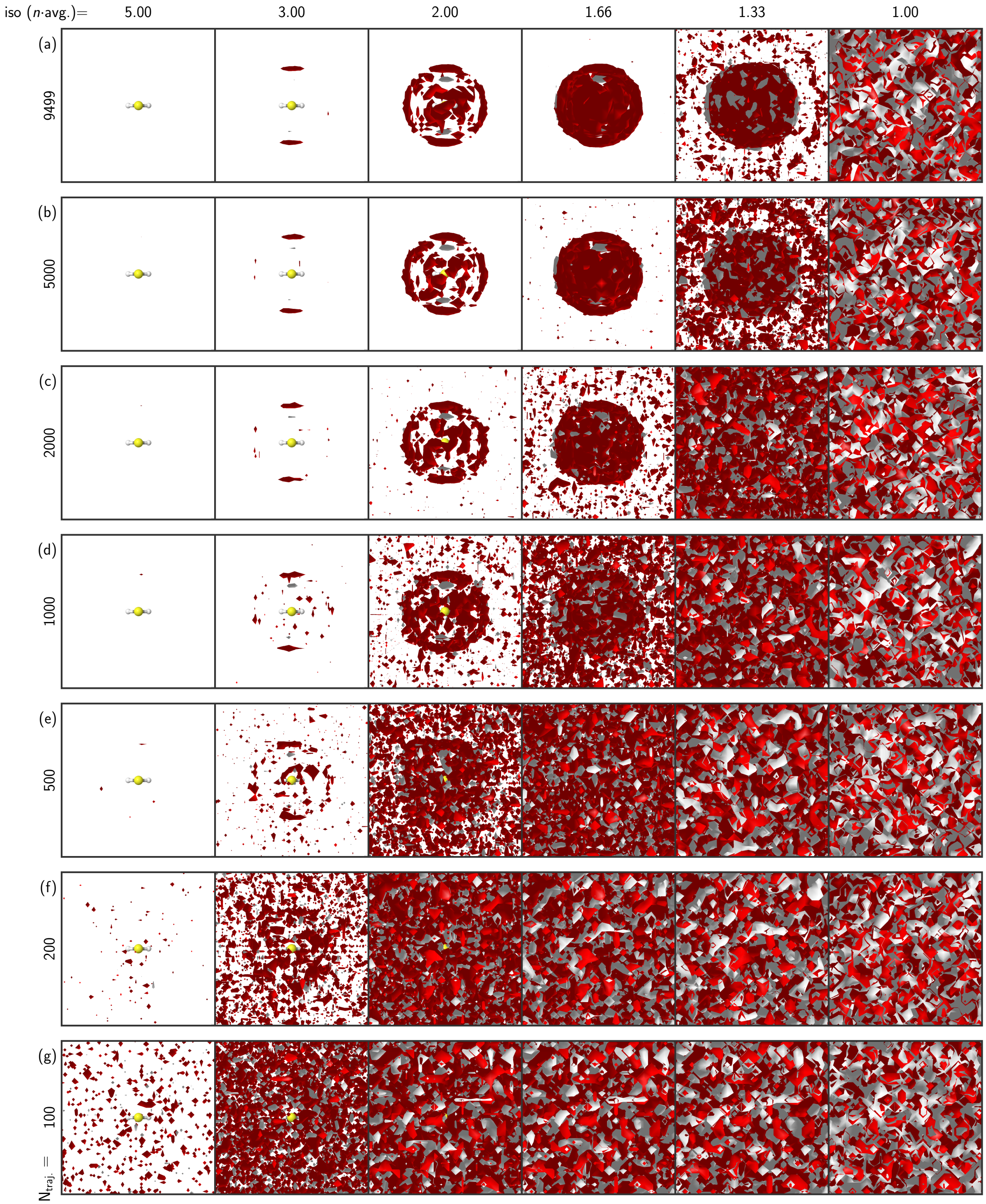

Figure S8: Comparison of time-dependent 3D-SDFs around CH_2S at t = 100 fs from the molecule's perspective for different isovalues for different numbers of trajectories. The different isovalues are given in multiples of the average number of water oxygen and hydrogen atoms, and the number of trajectories on the left of each panel.

S5 Electrostatic potentials

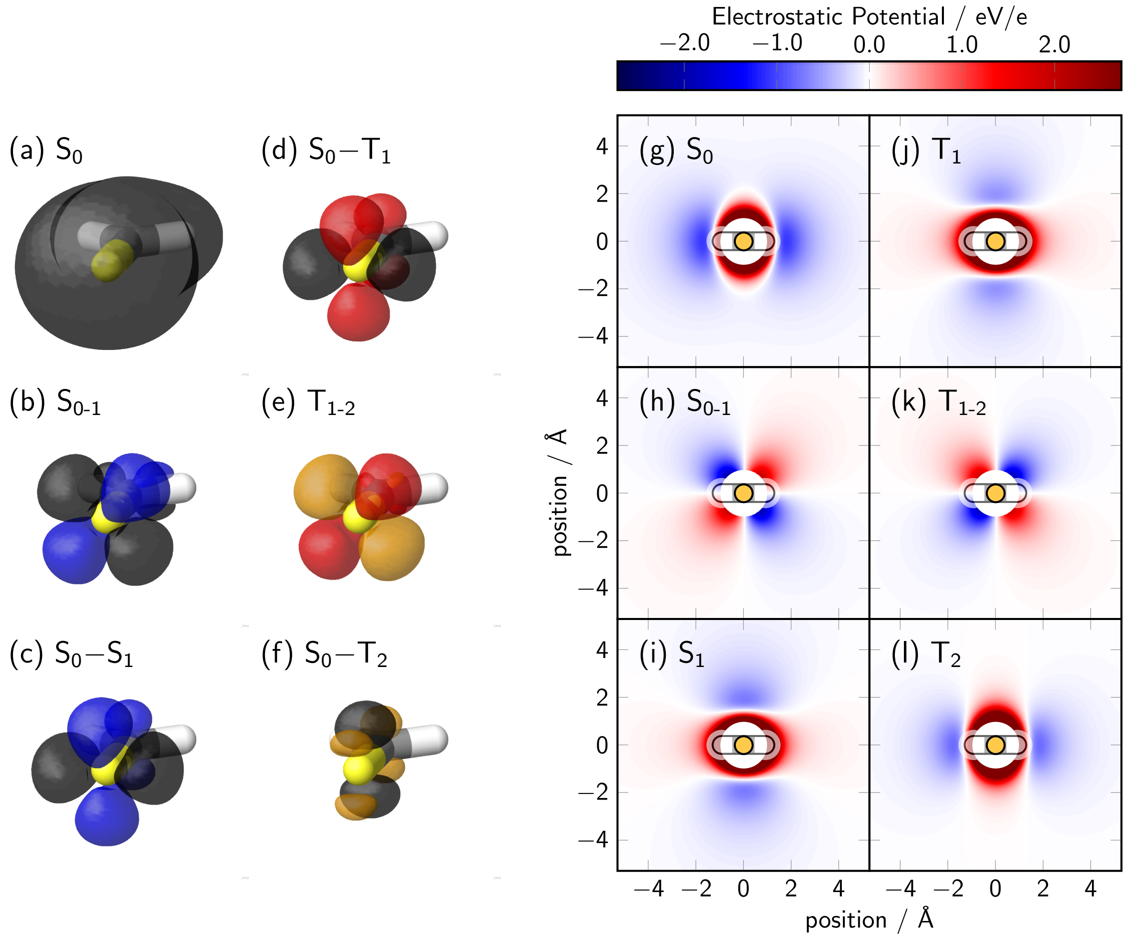

Figure S9: Electronic state and transition densities on the left, and electrostatic potential in the xy plane at the position of the sulfur atom of CH_2S for the electronic densities of all states and transition states on the right. Note that X_n is a electronic state density, X_{n-m} is a transition density and X_n-Y_m is a difference density. The colors black, blue, red and orange refer to S_0, S_1, T_1 and T_2, respectively. The densities are plotted with an isovalue of 0.01.

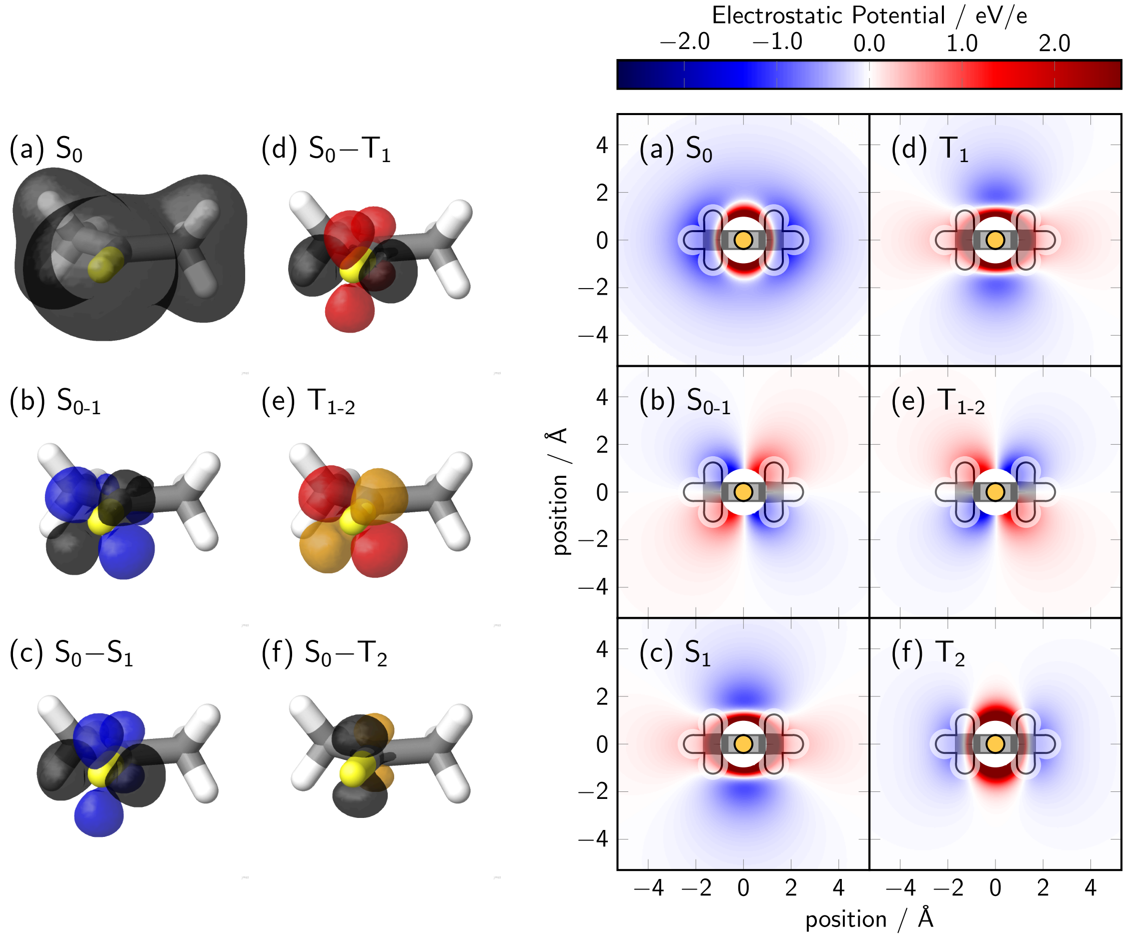

Figure S10: Electronic state and transition densities on the left, and electrostatic potential in the xy plane at the position of the sulfur atom of CMe_2S for the electronic densities of all states and transition states on the right. Note that X_n is a electronic state density, X_{n-m} is a transition density and $X_n{-}Y_m$ is a difference density. The colors black, blue, red and orange refer to S_0, S_1, T_1 and T_2, respectively. The densities are plotted with an isovalue of 0.01.

S6 Analysis of rotation around C-S axis

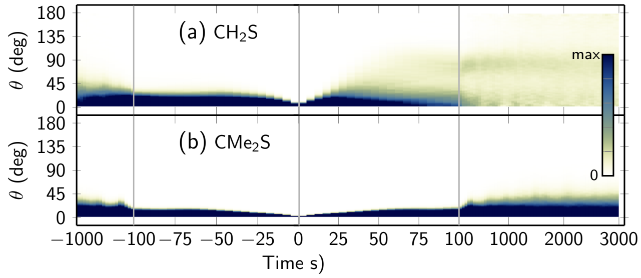

Figure S11: Relative rotation angle of the solute molecule over time. The rotation is calculated as the angle between the normal vector of the SCH_1 plane for CH_2S (a) and the normal vector of the $SC(C_{1,CH_3})$ plane for CMe_2S (b) at the current time and time 0.

S7 Temporal evolution of hydrogen bonds

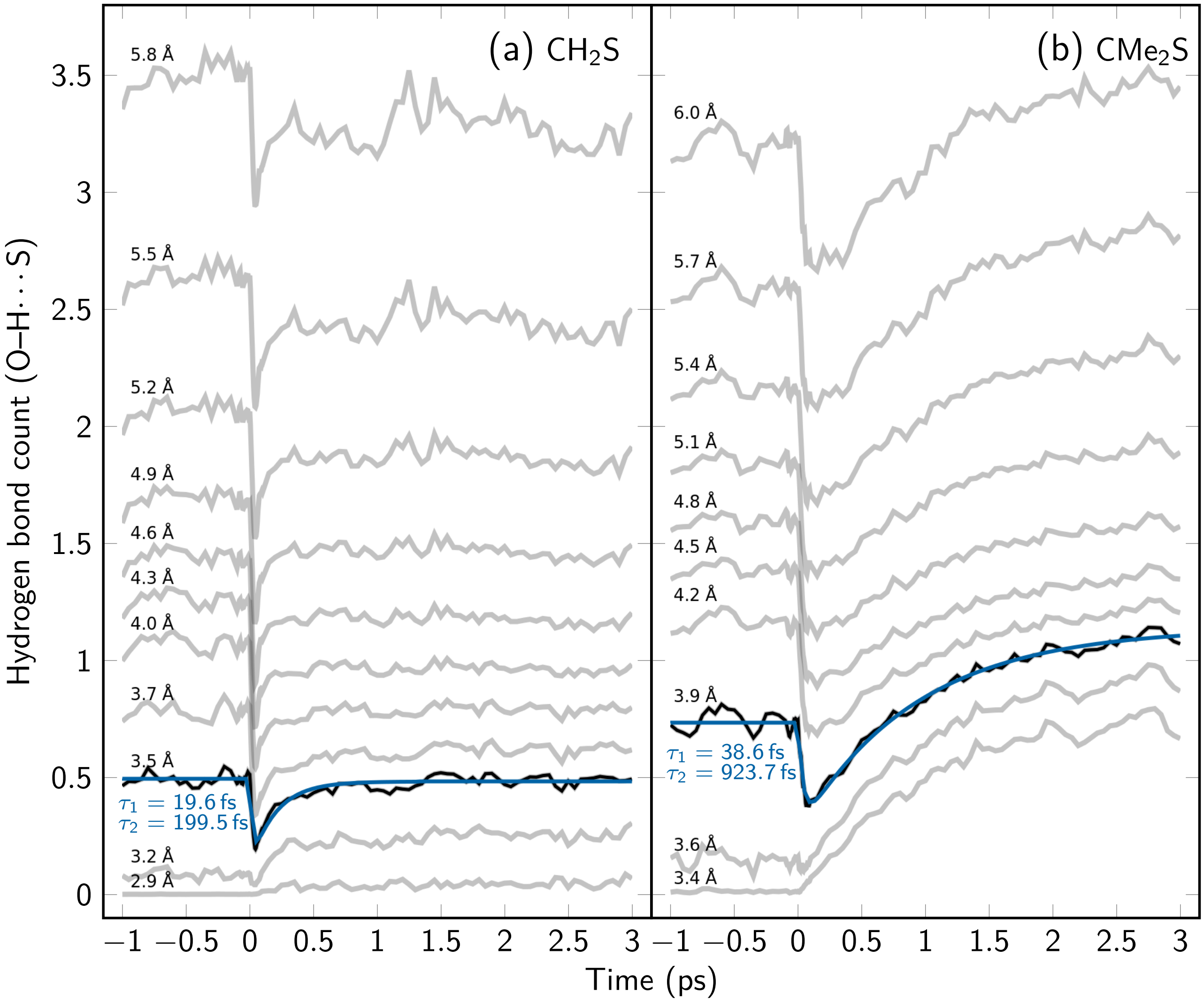

Figure S12: Temporal evolution of hydrogen bond count (O–H···S) for (a) CH_2S and (b) CMe_2S. Hydrogen bonds were counted using an O–H–S angle threshold of $\geq 135°$ and various O–S distance thresholds (indicated by the numbers left to each curve). The curves from the smallest distance thresholds that produced a significant drop at $t = 0\,\text{fs}$ (black curves) were selected for a biexponential fit with the function $f(t) = A + \Theta(t)(B - A)\left(1 + r\exp(-\frac{t}{\tau_1}) - (r+1)\exp(-\frac{t}{\tau_2})\right)$ (Θ is the Heaviside step function). The fits and obtained reorganization time constants are shown in blue.

S8 Shifts in eigenenergies of the coupled LVC Hamiltonian

Table S1: Shifted eigenenergies of the coupled LVC Hamiltonian matrix of for the thioformaldehyde and thioacetone systems at times 0 fs, 100 fs and 3000 fs. The energies are obtained by removing the energetic contribution of all solvent atoms and solvent-solvent interaction energies from the total system energy, taking the average, and removing the gas-phase energy of the solute at the equilibrium geometry.

State	CH_2S	CMe_2S
	time 0 fs	
S_0	-0.45 ± 0.18	-0.19 ± 0.15
S_1	0.17 ± 0.12	0.10 ± 0.13
T_1	0.20 ± 0.11	0.11 ± 0.13
T_2	-0.42 ± 0.23	-0.13 ± 0.20
	time 100 fs	
S_0	0.13 ± 0.10	0.08 ± 0.13
S_1	-0.27 ± 0.12	-0.08 ± 0.12
T_1	-0.19 ± 0.11	-0.05 ± 0.12
T_2	-0.38 ± 0.12	-0.23 ± 0.15
	time 3000 fs	
S_0	0.16 ± 0.12	0.14 ± 0.12
S_1	-0.29 ± 0.13	-0.41 ± 0.16
T_1	-0.22 ± 0.12	-0.34 ± 0.13
T_2	-0.25 ± 0.18	-0.15 ± 0.10

S9 Coherent Excitation of Normal Modes

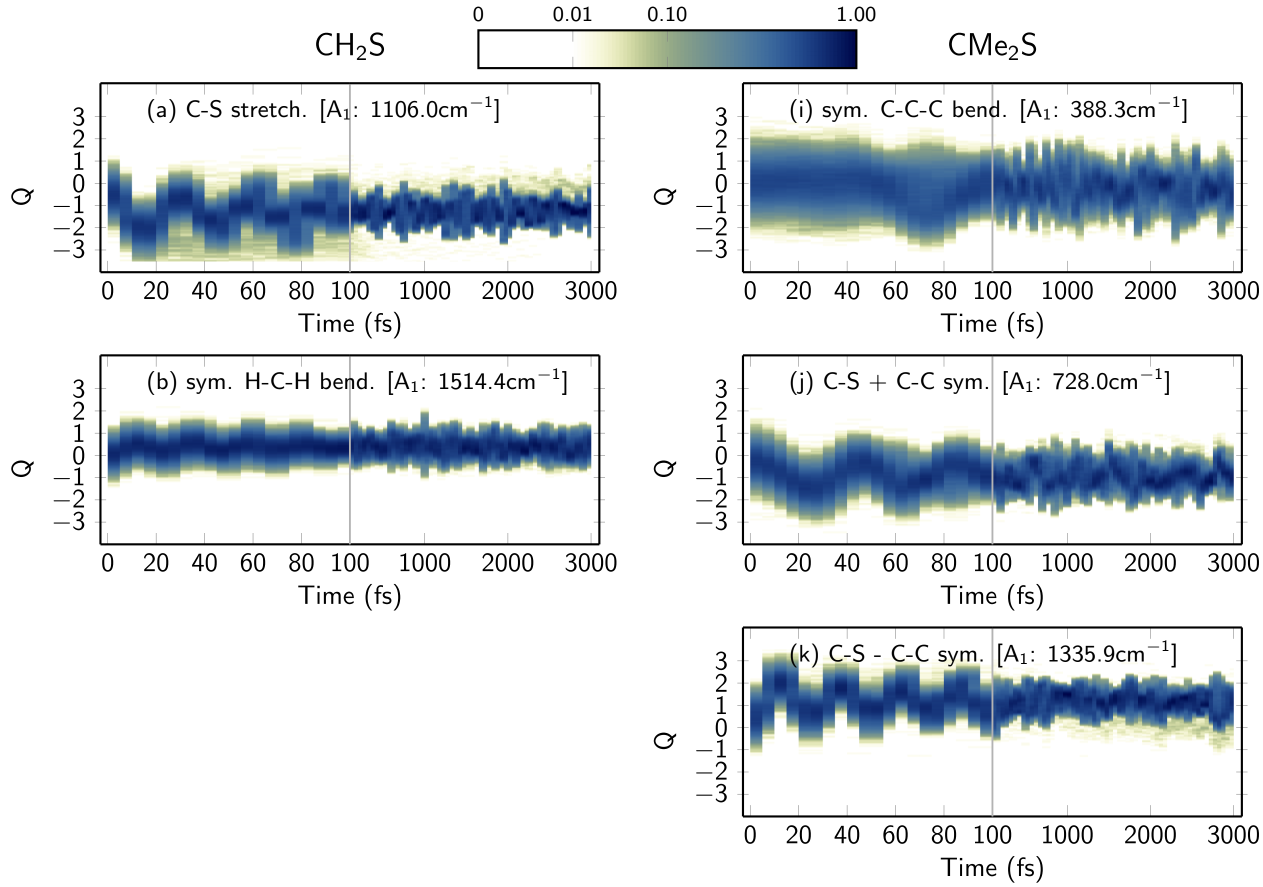

Figure S13: Convolutions of dimensionless mass–frequency-weighted normal modes coordinates (Q) over time for selected normal modes of CH_2S (left) and CMe_2S (right).

S10 Time-Dependent Couplings

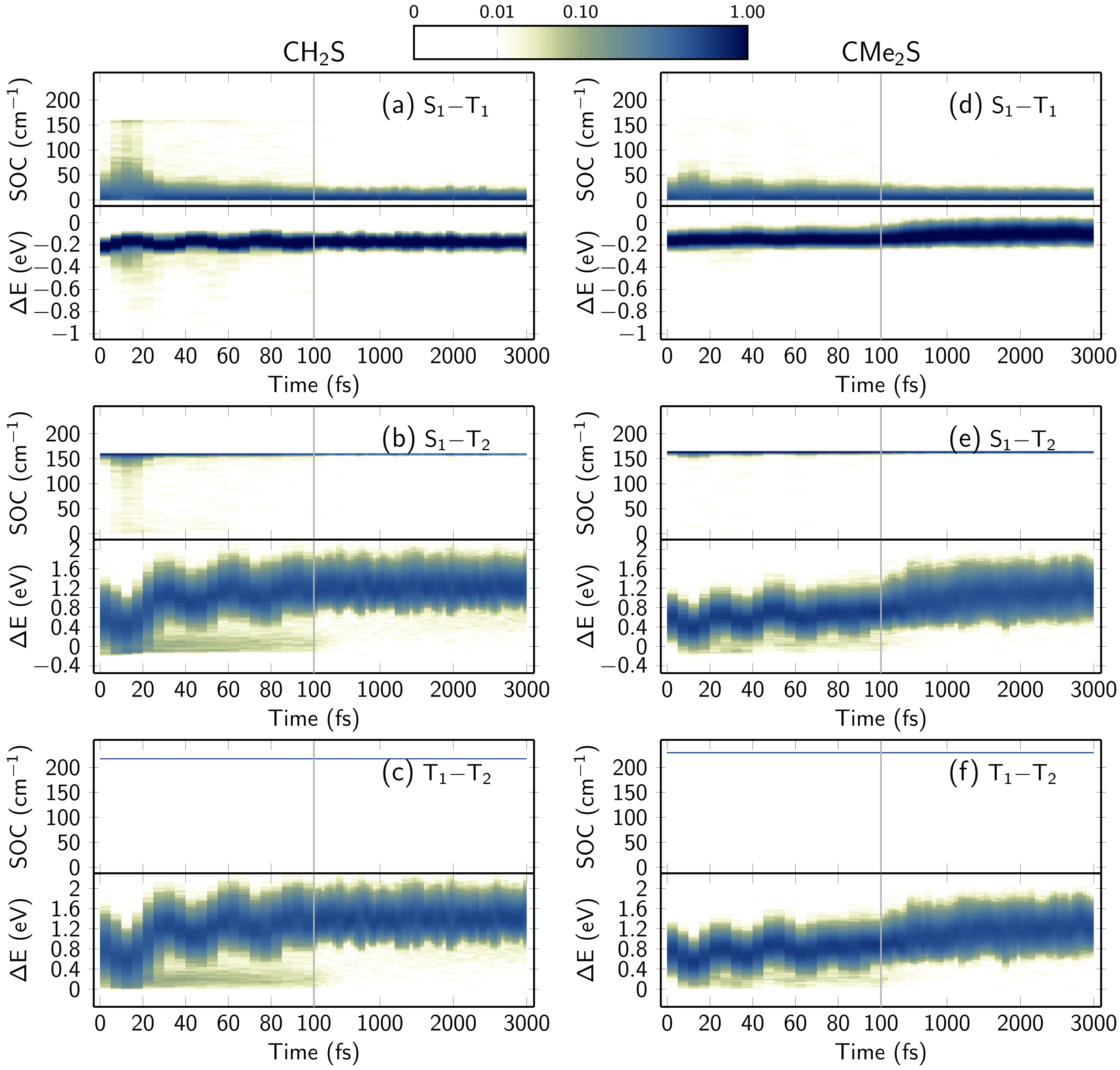

Figure S14: Convolutions of Spin-orbital couplings (SOCs) and energy gaps (ΔE) for all trajectories over time between different pairs of states for CH_2S (left) and CMe_2S (right).

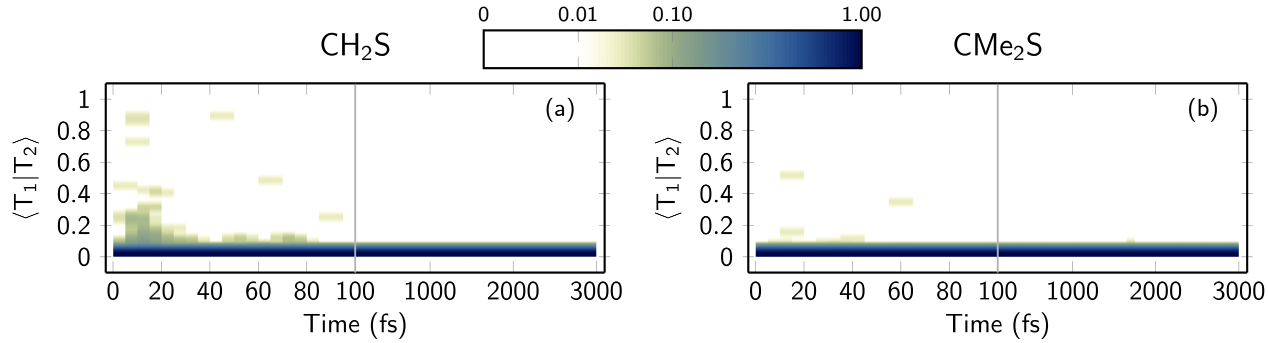

Figure S15: Time-dependent overlap between the T_1 and the T_2 state ($\langle T_1|T_2\rangle$) for CH_2S (left) and CMe_2S (right).
